# Wake‐Up and Fatigue under Electrical Cycling in HfO_2_‐Based Ferroelectrics: Mechanisms and Strategies toward Reliable Devices

**DOI:** 10.1002/advs.76737

**Published:** 2026-07-24

**Authors:** Hongseok Kim, Shinhyeong Lee, Hyojun Choi, Minseung Park, Hyunah Cho, Min Hyuk Park, Yunseok Kim

**Affiliations:** ^1^ School of Advanced Materials Science and Engineering Sungkyunkwan University (SKKU) Suwon Republic of Korea; ^2^ Department of Materials Science and Engineering College of Engineering Seoul National University Seoul Republic of Korea; ^3^ Department of Semiconductor and Display Engineering Sungkyunkwan University (SKKU) Suwon Republic of Korea; ^4^ Samsung Institute of Technology Samsung Electronics Co., Ltd. Samsung‐ro 1, Giheung‐gu Yongin‐si Gyeonggi‐do Republic of Korea; ^5^ Department of Materials Science and Engineering Inter‐University Semiconductor Research Center Research Institute of Advanced Materials, and Institute of Engineering Research College of Engineering Seoul National University Seoul Republic of Korea

**Keywords:** endurance, fatigue, HfO_2_‐based ferroelectrics, oxygen vacancy, wake‐up

## Abstract

HfO_2_‐based ferroelectrics have attracted intensive research interest as promising candidates for next‐generation nonvolatile memory, particularly due to their excellent complementary metal‐oxide‐semiconductor (CMOS) compatibility and scalability. Unlike conventional perovskite ferroelectrics, their ferroelectricity originates from a metastable non‐centrosymmetric phase, whose stability is highly sensitive to electrical cycling, defect chemistry, and processing conditions. Consequently, the evolution of ferroelectric properties during repeated electrical operation proceeds through two distinct phenomena, wake‐up and fatigue, whose combined progression determines device endurance and reliability. This review covers the physical origins and operating mechanisms of wake‐up and fatigue in HfO_2_‐based ferroelectrics, including oxygen vacancy dynamics and interfacial defect chemistry. Building on these insights, we discuss experimentally demonstrated strategies for controlling these behaviors, including composition and doping engineering, process optimization, electrode and interlayer design, and multilayer structural engineering. The discussion is further extended from materials‐level mechanisms to device‐level applications, with particular emphasis on how defect redistribution, charge trapping, domain‐wall pinning, and cycling‐induced phase evolution modify finite‐pulse switching kinetics. By correlating defect/phase‐transition physics, switching dynamics, and device‐level reliability metrics, this review provides design guidelines for the development of reliable HfO_2_‐based ferroelectric devices.

## Graphical abstract

HfO_2_‐based ferroelectrics exhibit wake‐up and fatigue behaviors during electrical cycling, significantly affecting device endurance and reliability. These phenomena are governed by defect dynamics, including oxygen vacancy redistribution and charge trapping. This review summarizes their physical origins and discusses engineering strategies to achieve reliable ferroelectric devices for scalable memory and computing applications.

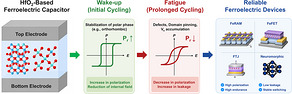

## 1 Introduction

Since ferroelectricity in HfO_2_ was first reported by Böscke et al. in 2011 [1], HfO_2_‐based ferroelectrics have emerged as one of the most intensively studied materials for next‐generation non‐volatile memories and integrated electronic devices [2, 3, 4]. Unlike conventional perovskite ferroelectrics such as BaTiO_3_ and SrBi_2_Ta_2_O_9_, which require complex integration schemes, and Pb(Zr,Ti)O_3_, which additionally raises concerns regarding Pb contamination and process compatibility, fluorite derived HfO_2_‐based materials offer several distinct advantages in terms of both material properties and semiconductor integration [5, 6]. These materials exhibit robust ferroelectricity even at thicknesses below 10 nm and excellent compatibility with complementary metal‐oxide‐semiconductor (CMOS) processing. They can be conformally deposited by atomic layer deposition, making them highly attractive for voltage scaling, integration in three dimensions, and applications oriented toward back‐end‐of‐line (BEOL) processes such as ferroelectric random‐access memory (FeRAM) and ferroelectric field effect transistors (FeFET) [7, 8, 9, 10, 11, 12, 13, 14, 15].

At the same time, the underlying physics of HfO_2_‐based ferroelectrics differs fundamentally from that of conventional perovskite ferroelectrics. Whereas perovskite systems typically derive ferroelectricity from a thermodynamically stable ferroelectric phase [16], ferroelectricity in HfO_2_‐based materials is generally associated with metastable polar phases rather than the equilibrium crystal structure [14, 17, 18, 19, 20]. In pristine HfO_2_, the thermodynamically favored phase is the centrosymmetric monoclinic phase. However, processing‐ and microstructure‐dependent factors, including mechanical stress, elemental doping, interfacial effects, oxygen vacancies, grain size reduction, and electric field application, can modify the free energy landscape and stabilize polar phases [21, 22, 23, 24, 25, 26, 27, 28]. Among these, the orthorhombic *Pca2_1_* phase has been studied most extensively as the primary origin of ferroelectricity [1, 17, 29], although additional polar polymorphs, including rhombohedral phases, have also recently been reported [30, 31, 32]. Consequently, the phase constitution and ferroelectric response of HfO_2_‐based ferroelectrics are highly sensitive to thermodynamic and kinetic boundary conditions, including processing history, defect configuration, and electrical stress [33, 34]. Because the ferroelectric phase is metastable, these integration advantages must be carefully considered together with reliability challenges such as wake‐up, fatigue, and imprint phenomena.

**FIGURE 1:**
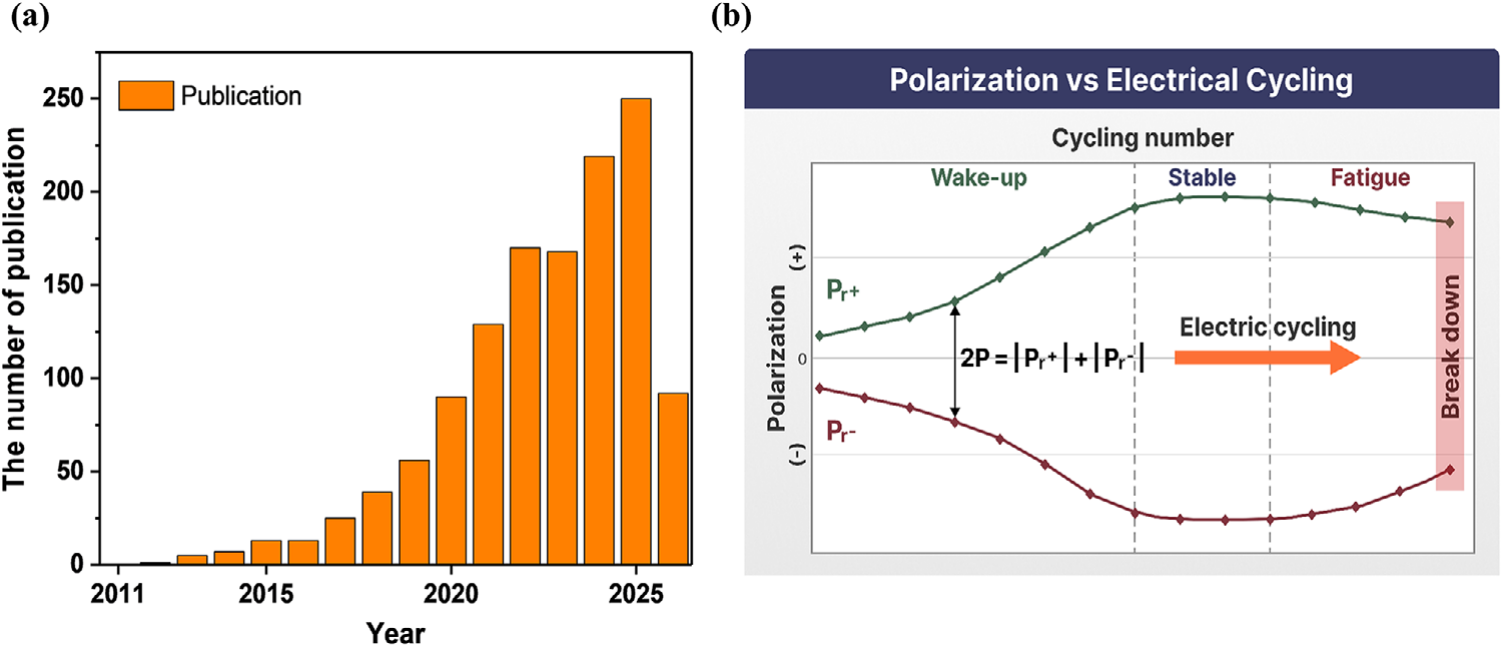
Research trends and electrical cycling behavior of HfO_2_‐based ferroelectrics. (a) Annual number of electrical cycling studies of HfO_2_‐based ferroelectrics over time. The data for 2026 was collected by June 30. (b) Evolution of *P_r_* as a function of electrical cycling, illustrating wake‐up, stable, and fatigue regimes prior to breakdown.

Accordingly, extensive efforts over the past decade have focused on inducing and optimizing ferroelectricity in HfO_2_‐based materials through control of composition, interfaces, microstructure, and processing conditions [35, 36, 37, 38, 39, 40]. However, as these strategies have developed, the central question has shifted from how to realize ferroelectricity to how that ferroelectricity evolves and survives during repeated operation. In other words, the evolution of ferroelectric properties with electrical cycling history has become a critical topic for both fundamental understanding and practical device reliability. As illustrated in Figure 1a, the number of studies on the electrical cycling response in HfO_2_‐based ferroelectrics has increased since the first observation of ferroelectricity in 2011, with rapid growth after the first observation of wake‐up behavior by Mueller et al. in 2013 [41]. This issue is particularly important because fluorite structured HfO_2_‐based ferroelectrics exhibit a distinctly non‐linear response to repeated electrical switching. Rather than showing only monotonic degradation, their cycling behavior is typically characterized by an initial wake‐up regime followed by fatigue at prolonged cycling, and the interplay between these regimes is related to endurance of the device, as schematically shown in Figure 1b. The wake‐up effect refers to the progressive enhancement of ferroelectric switching during early cycling, typically characterized by the opening of an initially pinched polarization‐electric field (P‐E) hysteresis loop and an increase in remanent polarization (*P_r_*). This cycling behavior contrasts with that of conventional perovskite ferroelectrics, for which the repeated electrical switching usually leads to a monotonic polarization fatigue without a wake‐up regime [42]. Since early studies on Si‐doped HfO_2_ identified this polarization enhancement under cyclic electrical stress [41, 43], extensive studies have examined its dependence on dopant composition, electrode structure, film thickness, and processing conditions [33, 44, 45]. However, with continued cycling, fatigue eventually develops, characterized by a reduction in *P_r_*, degraded switching characteristics, increased leakage current, and ultimately dielectric breakdown. These changes have been linked to electrically driven defect redistribution and accumulation, domain‐wall pinning, charge injection, and phase instability [46, 47]. Thus, endurance should be viewed as the cumulative outcome of the entire cycling trajectory from the pristine state through wake‐up and fatigue to failure.

From an application perspective, the additional cycling required to activate the full ferroelectric response increases operational complexity and can affect device reliability [48, 49]. At the same time, fatigue limits the long‐term stability required for memory and logic applications. Therefore, improving HfO_2_‐based ferroelectrics requires simultaneous suppression of undesirable wake‐up and retardation of fatigue, rather than addressing each phenomenon independently. The most desirable scenario is to achieve intrinsically stable ferroelectricity in the pristine state, i.e., wake‐up‐free or fast wake‐up behavior, while minimizing defect‐driven degradation, delaying breakdown, and extending endurance. Several excellent reviews have established the foundation of HfO_2_‐based ferroelectrics. Park et al. provided an early and influential overview of ferroelectricity and antiferroelectricity in doped HfO_2_‐based thin films, focusing on the emergence of polar and antipolar behavior, dopant‐dependent phase stabilization, and early memory‐related applications [5]. Chen et al. later offered a broad review of HfO_2_‐based ferroelectrics from phase structures and performance enhancement to material design, reliability challenges, and device applications [50]. In contrast to these broad‐scope reviews, the present review adopts electrical cycling reliability as its central organizing principle. Specifically, wake‐up, fatigue, imprint, leakage evolution, and dielectric breakdown are discussed within a unified mechanism‐driven framework based on defect kinetics, charge trapping, domain‐wall pinning, interfacial thermodynamics, cycling‐induced phase evolution, and leakage‐path formation. This perspective is particularly important because repeated electrical operation is where the metastable nature of HfO_2_‐based ferroelectric phases, oxygen‐vacancy dynamics, interface chemistry, and device failure intersect. Therefore, this review aims to bridge microscopic defect/phase‐transition physics with practical engineering strategies for wake‐up‐free, fatigue‐resistant, and high‐endurance HfO_2_‐based ferroelectric devices. Importantly, practical device reliability is determined not only by the *P_r_* retained after cycling, but also by how rapidly, completely, and reproducibly polarization can be reversed under finite programming pulses. Therefore, this review further places wake‐up and fatigue in the context of switching kinetics, including the evolution of switching‐current peaks, coercive field (*E_c_*) distributions, switching‐time distributions, finite‐pulse switched charge (*Q_sw_(t_p_)*), and positive/negative switching asymmetry.

This review also incorporates several aspects that have received less attention in previous works. Previous studies on electrical cycling behavior have primarily focused on the ferroelectric orthorhombic (*Pca2_1_*) phase. In contrast, this review additionally incorporates recent advances on the rhombohedral phase, which exhibits distinct polarization formation mechanisms and weak or nearly wake‐up‐free behavior [30, 31], providing additional insight into the differences in phase stability and cycling behavior between orthorhombic and rhombohedral phases. In addition, recent studies addressing the practical impact of wake‐up and fatigue on device‐ and circuit‐level operation, including FeRAM, FeFET, ferroelectric tunnel junction (FTJ), and emerging neuromorphic and computing‐in‐memory architectures, are also discussed.

## 2 Wake‐Up and Fatigue Phenomena

To move beyond reported wake‐up and fatigue phenomena, this review adopts a mechanism‐driven framework for electrical cycling reliability in HfO_2_‐based ferroelectrics. The discussion is organized around four coupled mechanisms: defect kinetics and oxygen‐vacancy redistribution, charge trapping and domain‐wall pinning, interfacial thermodynamics and cycling‐induced phase evolution, and leakage‐path formation leading to dielectric breakdown. Within this framework, wake‐up is treated as a non‐equilibrium kinetic activation process, whereas fatigue is treated as a longer‐term degradation process in which defect kinetics become coupled with the thermodynamic stability of metastable polar phases at ferroelectric/electrode interfaces. This approach allows reported experimental observations to be interpreted as evidence for specific microscopic mechanisms rather than as isolated case studies.

### 2.1 Definition of Wake‐Up and Fatigue Behavior

The wake‐up phenomenon in HfO_2_‐based ferroelectrics refers to the progressive opening of an initially pinched P‐E hysteresis loop, accompanied by an increase in *P_r_*, during repeated electrical cycling from the pristine state [8, 22, 41, 43, 51, 52]. This wake‐up behavior has been widely reported across various materials. For example, Zhou et al. reported that Si‐doped HfO_2_ thin films exhibit wake‐up behavior strongly dependent on the amplitude and frequency of the applied electric field [43]. Starschich et al. investigated the influence of electrode configuration on the wake‐up phenomenon in Y‐doped HfO_2_ thin films and highlighted the role of oxygen vacancy redistribution [53]. Song et al. further analyzed the effects of film thickness and La doping concentration on the wake‐up behavior in epitaxial La‐doped Hf_0.5_Zr_0.5_O_2_ (HZO) ferroelectric thin films [54].

In contrast, under prolonged electrical cycling, the ferroelectric properties gradually deteriorate. This phenomenon is referred to as fatigue. In general, fatigue is characterized by a decrease in *P_r_* and the reappearance of pinched or increasingly asymmetric hysteresis loops as cycling proceeds [55, 56]. Numerous studies have identified defect generation, particularly the formation of oxygen vacancies, as a major factor contributing to fatigue [46, 57]. The accumulation of such defects can lead to local electric field concentration, which in turn can increase leakage current and promote the formation of conductive paths during prolonged electrical cycling [21]. In addition, charge injection and domain‐wall pinning have been reported as important mechanisms contributing to fatigue behavior [8, 46, 58]. Notably, fatigue cannot be attributed to a single mechanism but arises from the combined effects of multiple factors. Therefore, understanding the microscopic origin of fatigue is essential for improving the endurance and long‐term reliability of HfO_2_‐based ferroelectric devices. In the following section, we discuss these mechanisms in HfO_2_‐based ferroelectrics, particularly focusing on the role of oxygen vacancies and defect redistribution during electrical cycling.

In addition to quasi‐static hysteresis parameters, wake‐up and fatigue should also be interpreted in terms of polarization‐switching kinetics under finite electric‐field pulses. Here, switching kinetics refers to the field‐ and time‐dependent polarization‐reversal process, including domain nucleation, domain wall motion, switching‐current evolution, *E_c_* distribution, local switching‐time distribution, and *Q_sw_(t_p_)*. Classical ferroelectric switching has often been discussed using Kolmogorov–Avrami–Ishibashi (KAI)‐type descriptions, which emphasize nucleation and domain‐growth‐mediated switching, and nucleation‐limited‐switching (NLS)‐type descriptions, which describe polarization reversal through distributed local switching times. For polycrystalline HfO_2_‐based ferroelectrics, such a distributed‐switching picture is useful because grain‐to‐grain structural variation, phase coexistence, oxygen‐vacancy distribution, trapped charges, and internal‐bias fields can broaden local switching‐field and switching‐time distributions. Therefore, wake‐up and fatigue should be viewed not only as changes in *P_r_*, but also as cycling‐induced evolution of finite‐pulse switching dynamics [59, 60].

Recent pulse‐switching and nucleation‐limited‐switching analyses provide direct experimental support for this defect‐controlled switching‐kinetics picture [61]. In atomic layer deposition (ALD)‐grown HZO films, residual impurities such as C‐ and N‐related defects were shown to modify both microstructure and polarization reversal: increased impurity concentration reduced the grain size, shortened the median switching time, and broadened the switching‐time distribution. This indicates that impurities and grain boundaries can act as heterogeneous nucleation sites that lower local switching barriers, while simultaneously increasing spatial inhomogeneity in the nucleation landscape. Similarly, pulse‐switching measurements of HZO wake‐up showed that field cycling depins initially pinned or anti‐parallel domains and promotes the conversion of non‐ferroelectric interfacial regions into ferroelectrically active regions. Therefore, defect redistribution during wake‐up can activate and homogenize finite‐pulse polarization reversal, whereas excessive defect generation, trapped charge, and defect accumulation during fatigue broaden the local switching‐barrier distribution and degrade deterministic switching.

### 2.2 Origins of Wake‐Up Behavior

**FIGURE 2:**
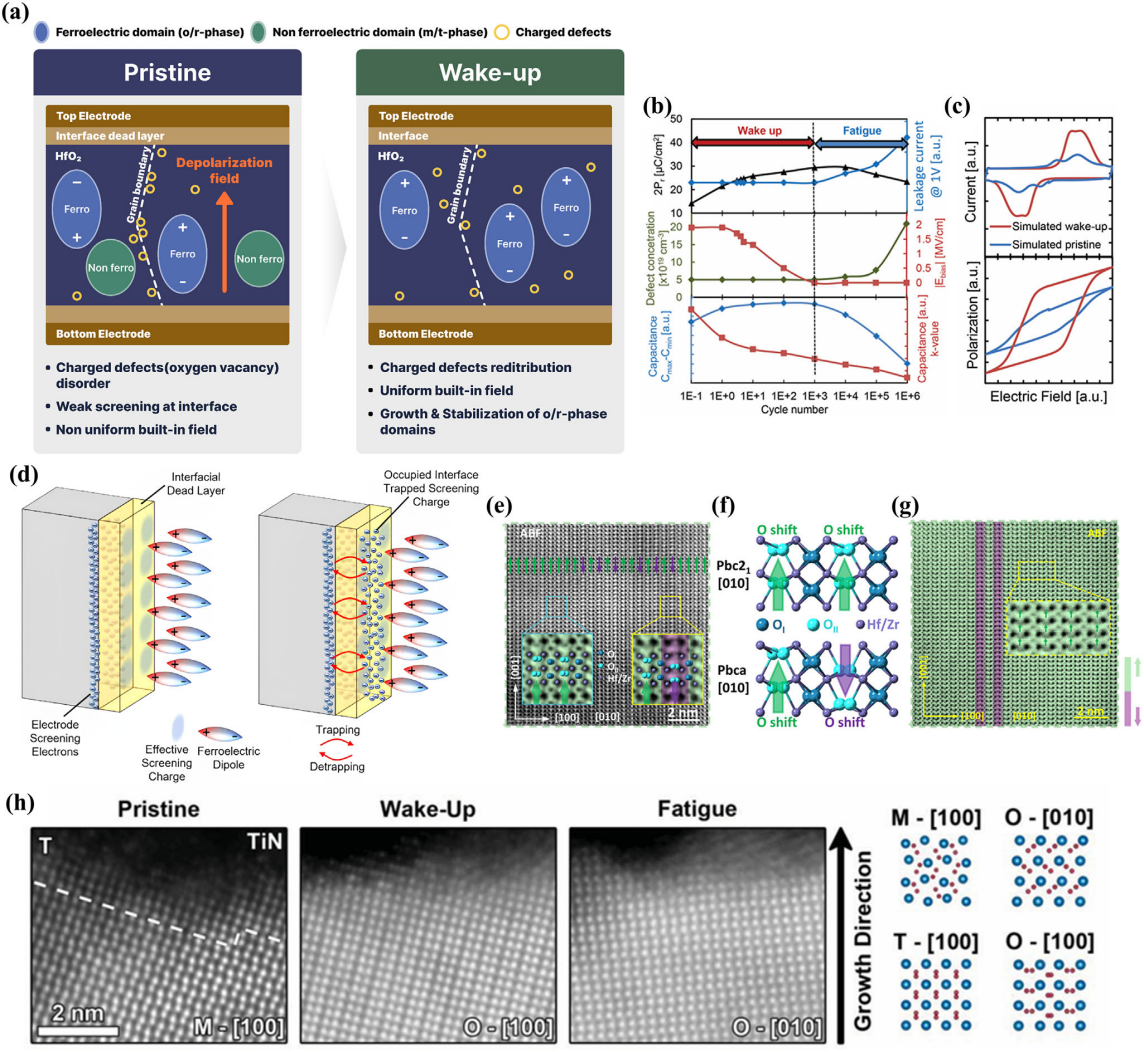
Wake‐up mechanisms in HfO_2_‐based ferroelectrics. (a) Schematic of the wake‐up mechanism in HfO_2_‐based ferroelectric capacitors during electrical cycling, highlighting defect dynamics, interfacial screening, and domain evolution. (b) Evolution of *P_r_* and leakage current, along with extracted defect concentration, bias field, and capacitance, during bipolar electrical cycling in Sr:HfO_2_‐based ferroelectric capacitors. (c) Simulated resulting I‐V and P‐V characteristics obtained by removing the charges from the interface. Reproduced with permission [46]. Copyright 2016, WILEY‐VCH Verlag GmbH & Co. KGaA, Weinheim. (d) Depolarization field reduction via charge injection and trapping at the electrode–ferroelectric interface during wake‐up. Reproduced with permission [62]. Copyright 2020, American Chemical Society. (e‐g) ABF analyses of TiN/HZO/TiN ferroelectric films. (e) ABF image of pristine 15 nm HZO film showing oxygen atomic columns with distinct displacement offsets and directions. Based on the displacement of oxygen columns relative to the center of the four nearest Hf/Zr columns, the oxygen sites are classified into O_I_ (centered) and O_II_ (off‐centered) types, where the O_II_ columns exhibit shifts along both the [001] and opposite directions. (f) Atomic models of the ferroelectric *Pbc2_1_* and anti‐ferroelectric *Pbca* phases along the [010] direction, showing centered and off‐centered oxygen configurations within the Hf/Zr lattice. (g) ABF analysis of the TiN/HZO/TiN device after wake‐up induced by bipolar triangular pulses (±4 V, 10^4^ cycles, f = 500 kHz), showing the transformation from the *Pbca* phase to the *Pbc2_1_* phase. The majority of off‐centered O_II_ oxygen atomic columns are displaced along the [001] direction, indicating formation of a *Pbc2_1_*‐dominated ferroelectric structure. Reproduced with permission [63]. Copyright 2022, Springer Nature Limited. (h) STEM HAADF images of Gd‐doped HfO_2_ in pristine, wake‐up and fatigue conditions, showing relaxation of the bulk monoclinic (paraelectric) and tetragonal (paraelectric) symmetry at the electrode interfaces toward orthorhombic (ferroelectric) phase, a trend which is most pronounced in the pristine and least pronounced in the fatigued. Image levels adjusted to enhance contrast. Reproduced with permission [46]. Copyright 2016, WILEY‐VCH Verlag GmbH & Co. KGaA, Weinheim.

The underlying mechanism of the wake‐up phenomenon is widely understood to originate from the interplay between ferroelectric and dielectric contributions as shown in Figure 2a. Specifically, ferroelectric properties, such as domain reorientation, phase stabilization, and field‐induced phase transitions, interact with dielectric‐related processes, including defect formation and charge trapping, forming a complex electrical environment [46, 64]. During electrical cycling, the built‐in electric field is gradually modified, which stabilizes the ferroelectric phases and reconstructs the domain structure, leading to the emergence of wake‐up behavior [53, 60].

Among the various proposed mechanisms, the wake‐up behavior is commonly attributed to the migration and redistribution of oxygen vacancies coupled with structural transition from non‐ferroelectric phases to the ferroelectric orthorhombic phase, as shown in Figure 2a. Under a high electric field, charged oxygen vacancies become highly mobile and undergo field‐driven drift and redistribution on relatively short time scales [65]. This non‐equilibrium defect dynamics modifies the local free energy landscape and lowers the kinetic barriers for phase transitions, thereby facilitating the stabilization of the ferroelectric orthorhombic phase from non‐polar tetragonal or monoclinic phases [66]. Therefore, the phase transition observed during wake‐up should be understood as a kinetically driven process enabled by electric field‐assisted defect migration and defect‐mediated polymorphism kinetics assisted by the electric field, rather than a transition toward thermodynamic equilibrium. Providing comprehensive evidence for these mechanisms, Pešić et al. investigated the electrical cycling behavior of HfO_2_‐based ferroelectric capacitors by combining electrical characterization, microstructural observations, and numerical modeling [46]. Their study clearly distinguished different wake‐up regimes using polarization hysteresis, switching current analyses, Preisach density analysis, leakage current spectroscopy, and transmission electron microscopy (TEM) observations. They showed that the increase in *P_r_* during wake‐up originates primarily from the redistribution of pre‐existing oxygen vacancies rather than from the generation of new defects. This interpretation is supported by the observation that the leakage current remains nearly unchanged over the initial cycling regime, as shown in Figure 2b. During electrical cycling, field‐driven diffusion and recombination of oxygen vacancies and oxygen ions gradually reduce the initially non‐uniform built‐in field. This redistribution leads to a more homogeneous internal electric field, allowing a larger fraction of domains to participate in polarization switching and resulting in the de‐pinching of the hysteresis loop. These mechanisms are supported by quantitative agreement between experimental measurements and numerical simulations, including technology computer aided design (TCAD) and drift‐diffusion models that account for defect diffusion and electric field distribution. As shown in Figure 2b,c, both experimental and simulation results consistently reproduce the relaxation of the internal built‐in field and the corresponding evolution of hysteresis loop. Leakage current analysis further suggests that conduction is dominated by oxygen vacancies located at grain boundaries, explaining why the overall leakage current remains relatively unchanged despite defect redistribution. Experimental observations also show that increasing temperature enhances oxygen vacancy diffusion, thereby accelerating the wake‐up process. This behavior is consistent with reports that TiN electrodes undergo partial oxidation during electrical operation and act as sinks for oxygen vacancy, thereby influencing both oxygen vacancy concentration and internal electric field distribution [67].

From a switching‐kinetics perspective, wake‐up corresponds to the progressive activation and homogenization of polarization reversal. Redistribution of pre‐existing oxygen vacancies relaxes the initially nonuniform internal‐bias field and reduces local domain pinning, thereby narrowing the distribution of effective switching fields and switching times. As a result, regions that are initially pinned, back‐switched, or kinetically inactive can participate in polarization reversal within a given pulse duration. Therefore, wake‐up produces not only an increase in *P_r_* and de‐pinching of the hysteresis loop, but also more complete, faster, and more symmetric switching under finite‐pulse operation.

This interpretation is consistent with pulse‐switching studies of HZO capacitors, where the pristine state contained pinned or anti‐parallel ferroelectric domains together with a non‐ferroelectric tetragonal‐phase contribution [19]. After field cycling, the pinned domains were largely depinned and the interfacial non‐ferroelectric phase was partially converted into the ferroelectric phase, as inferred from the increase in interfacial capacitance and switched polarization. These results indicate that wake‐up increases not only the amount of switchable polarization but also the fraction of domains that can respond within a finite pulse, thereby improving *Q_sw_(t_p_)*, switching symmetry, and switching reproducibility.

Further supporting this defect‐driven interpretation, Starschich et al. investigated the wake‐up phenomenon in Y‐doped HfO_2_ thin films through frequency‐ and temperature‐dependent electrical cycling experiments [68]. Their results demonstrated that wake‐up behavior depends primarily on the duration of the applied electric field rather than the number of cycles and exhibits strong thermally activated characteristics, providing further evidence that ion migration governs the process. In addition, observations of resistive switching behavior in asymmetric electrode structures supported the conclusion that oxygen vacancy migration and redistribution are key factors in wake‐up.

From a complementary perspective focusing on the interfaces, Lomenzo et al. proposed that the wake‐up phenomenon can be interpreted in terms of changes in the depolarization field [62]. The depolarization field, arising from incomplete screening at the electrodes and the presence of non‐ferroelectric interfacial layers, suppresses polarization switching in the pristine state by inducing back‐switching and resulting in a pinched hysteresis loop. As electrical cycling proceeds, as shown in Figure 2d, interfacial screening improves through charge injection and trapping, leading to a gradual reduction of the depolarization field. Consequently, polarization switching becomes more stable, and the wake‐up behavior emerges.

Although these defect‐driven and interfacial interpretations are strongly supported by electrical measurements and modeling, direct experimental visualization of oxygen vacancy migration remains challenging. To date, no studies have directly tracked oxygen vacancy motion using scanning transmission electron microscopy (STEM)‐based analyses. Nevertheless, several studies have provided indirect evidence by observing structural changes induced by electrical cycling, particularly phase transitions from non‐polar phases to orthorhombic ferroelectric phases. For example, Cheng et al. used atomic‐resolution Cs‐corrected annular bright‐field (ABF) images to show that the wake‐up in HZO thin films is associated with a reversible phase transition from the non‐polar antiferroelectric *Pbca* phase to the polar ferroelectric *Pca2_1_* phase (denoted as *Pbc2_1_* in their work to maintain axis consistency with the *Pbca* phase) as shown in Figure 2e–g [63]. Electrical cycling induces the local displacement of oxygen ions, leading to an increased fraction of the ferroelectric phase, consistent with the observed increase in *P_r_* without a corresponding increase in leakage current. Similarly, high‐angle annular dark‐field (HAADF) analysis of Gd‐doped HfO_2_ thin films confirmed that the fraction of the monoclinic phase decreases while the fraction of the ferroelectric orthorhombic phase gradually increases with electric cycling as shown in Figure 2h [46]. In addition, the degree of lattice relaxation along the tetragonal symmetric direction near the electrode interface decreases with cycling, indicating progressive stabilization of the ferroelectric orthorhombic phase under electrical driving.

By contrast, most existing studies have explained the wake‐up mechanism primarily on the basis of the ferroelectric orthorhombic phase. However, since the ferroelectric rhombohedral phase was first reported in 2018 [30], interest in its wake‐up characteristics has gradually increased. Notably, several recent studies have reported that the rhombohedral phase exhibits little or negligible wake‐up behavior compared with the orthorhombic phase [31, 69, 70, 71]. Density functional theory (DFT)‐based analyses have further suggested that the rhombohedral phase is an intrinsically ferroelectric phase with stable polarization in the pristine state [72]. In other words, unlike the orthorhombic phase, it does not require a transition from a non‐polar phase into a polar ferroelectric phase, and electrical cycling primarily enhances the stability and symmetry of polarization switching. These differences originate from fundamentally distinct polarization formation mechanisms. In the orthorhombic phase, ferroelectricity generally emerges through electric‐field‐induced phase transitions and defect redistribution involving non‐polar phases such as tetragonal or monoclinic structures. In contrast, polarization in the rhombohedral phase intrinsically arises from spontaneous symmetry breaking within the fluorite lattice, frequently stabilized by strain effects.

Nevertheless, the rhombohedral phase is not entirely independent of electrical cycling, and its weak wake‐up behavior can still be interpreted through two primary mechanisms [72]. First, similar to the wake‐up behavior of the orthorhombic phase, redistribution of charged defects, particularly oxygen vacancies, reduces domain pinning and improves the symmetry of polarization switching in pre‐existing ferroelectric domains. Second, residual non‐polar tetragonal (*P4_2_/nmc*) phase can partially transform into the polar rhombohedral (*R3*) phase under an applied electric field, thereby increasing the fraction of switchable polarization. In particular, the tetragonal phase is considered to act as a saddle point within the free‐energy landscape, enabling transition into the rhombohedral phase through a relatively small energy barrier.

Despite these advances, the wake‐up‐free or weak wake‐up characteristics in the rhombohedral phase have only recently been reported, and additional experimental and theoretical studies are still necessary to establish relationships among oxygen vacancy dynamics, phase stability, and polarization switching. Therefore, the following discussion on wake‐up and fatigue mechanisms, as well as related material and device design strategies, primarily focuses on the orthorhombic phase.

### 2.3 Mechanisms of Fatigue in HfO_2_‐Based Ferroelectrics

**FIGURE 3:**
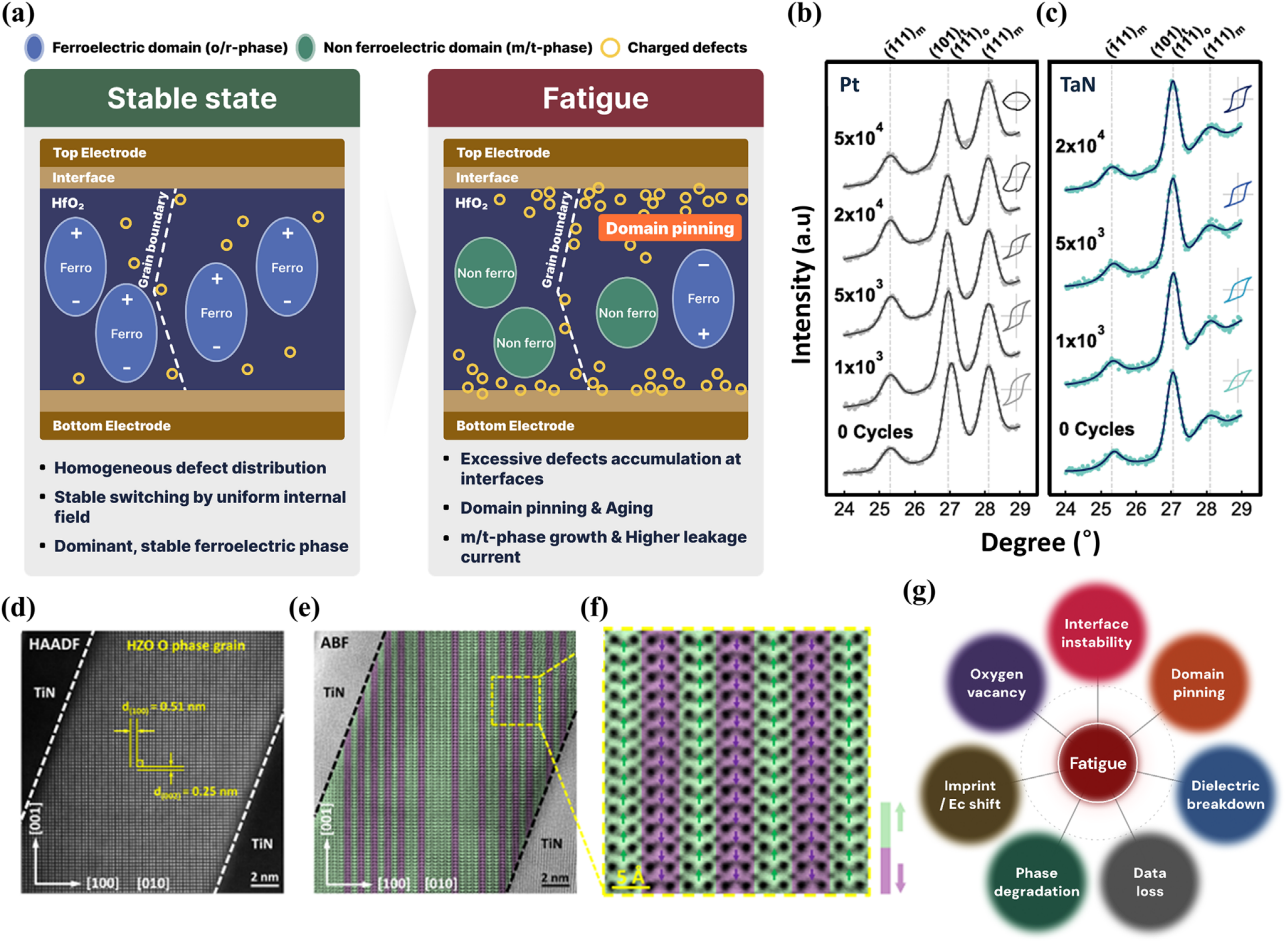
Fatigue mechanisms and effect in HfO_2_‐based ferroelectrics. (a) Schematic illustration of the progressive accumulation of charged defects and associated domain pinning during electrical cycling. (b, c) Synchrotron XRD patterns and corresponding P‐E hysteresis loops at successive cycling intervals for symmetric MIM Hf_0.58_Zr_0.42_O_2_ capacitors with (b) Pt and (c) TaN electrodes. Reproduced with permission [73]. Copyright 2020, American Chemical Society. (d) Cross sectional STEM HAADF image of a fatigued TiN/HZO/TiN capacitor showing an orthorhombic phase grain along the [010] zone axis. (e) Corresponding ABF image showing alternating displacement of off‐center oxygen columns along the [001] direction (green) and its antiparallel direction (purple), characteristic of the antipolar *Pbca* phase. (f) Magnified ABF image of the boxed region in (e), with arrows indicating the oxygen displacement directions relative to the surrounding Hf/Zr sites. Reproduced with permission [63]. Copyright 2022, Springer Nature Limited. (g) Key factors and phenomenon contributing to fatigue, including defect accumulation, domain pinning, interfacial instability, and breakdown.

While fatigue in conventional perovskite ferroelectrics has been extensively studied, with domain‐wall pinning, charge injection, and interfacial defect accumulation identified as key contributing mechanisms [42, 74, 75, 76, 77, 78], fatigue in HfO_2_‐based ferroelectrics shares some of these features but is further complicated by cycling‐induced phase transitions between polar and non‐polar phases. Building on the defect dynamics discussed for the wake‐up mechanism, oxygen vacancies continue to play a crucial role in governing the electrical behavior of HfO_2_‐based ferroelectrics [6, 46, 79, 80]. During repeated electrical cycling, these positively charged defects can migrate under the applied electric field and gradually redistribute within the ferroelectric layer, preferentially accumulating near interfaces and grain boundaries as shown in Figure 3a [81, 82, 83]. Strong local electric fields generated during domain nucleation and growth further promote the drift and diffusion of oxygen vacancies, continuously modifying the internal field distribution throughout the cycling process [64, 68, 84].

Expanding on this defect migration process, Pešić et al. demonstrated how the dynamic evolution of oxygen vacancies determines the full lifecycle of HfO_2_‐based ferroelectric capacitors during electrical cycling [46]. During the wake‐up stage, the field‐driven redistribution of pre‐existing vacancies leads to a more homogeneous defect distribution and reduces the built‐in field. However, under prolonged cycling, additional oxygen vacancies are generated in both bulk and interfacial regions, increasing the overall defect density [85]. These newly generated oxygen vacancies act as charge trapping centers, locally distorting the internal electric field and promoting domain pinning. As a result, polarization switching becomes increasingly hindered, leading to a reduction in *P_r_* and the onset of fatigue. In this regard, the evolution of oxygen vacancies and associated charge trapping are considered to play a critical role in the fatigue behavior.

In addition to these defect dynamics, fatigue can also arise from structural evolution of the ferroelectric phase itself. Fields et al. provided crystallographic evidence using synchrotron X‐ray diffraction (XRD), showing that degradation of *P_r_* is closely linked to phase transformation during cycling [73]. Specifically, prolonged electrical cycling induces systematic changes in phase fractions, with the relative proportions of polar and non‐polar phases depending on the electrode material. As shown in Figure 3b, for Pt electrodes, the overlapped tetragonal/orthorhombic reflection near 27° in 2*θ* progressively decreased in intensity with cycling, while the monoclinic reflections grew, indicating that fatigue in Pt electrode capacitors originates from an irreversible conversion of the polar orthorhombic and non‐polar tetragonal phases to the non‐polar monoclinic phase, accompanied by a collapse of the corresponding P‐E hysteresis loop. In contrast, when reactive electrodes such as W or TaN are employed, the same cycling conditions produced wake‐up rather than fatigue, with a progressive increase in *P_r_* and evolution of the P‐E hysteresis loop from a pinched to a saturated shape. As shown in Figure 3c, for TaN electrodes, the monoclinic and tetragonal/orthorhombic phase peak intensities remained unchanged during cycling, while the tetragonal/orthorhombic phase peak narrowed and shifted slightly to larger d‐spacings. This structural change indicates that the polarization enhancement in this case is driven by a partial conversion from the non‐polar tetragonal to the polar orthorhombic phase, rather than a suppression of the monoclinic phase.

The underlying mechanism governing this distinct phase evolution heavily depends on oxygen exchange and interfacial redox chemistry at the boundary between the ferroelectric and the metal. Noble metal electrodes such as Pt are relatively inert and have limited oxygen‐exchange capability; therefore, in some HfO_2_‐based stacks, they may limit defect compensation and facilitate cycling‐induced relaxation of the metastable polar orthorhombic/tetragonal phase fraction toward the non‐polar monoclinic phase during prolonged electrical stress. In contrast, reactive electrodes such as W, TiN, and TaN can participate in partial oxidation or oxynitride formation, thereby modifying the local oxygen chemical potential and oxygen‐vacancy concentration at the ferroelectric/metal interface. Interfacial reaction layers such as WO*_x_*, TiO*_x_*, TiON, and TaON can act as oxygen reservoirs, oxygen sinks, or defect‐generation sources depending on their oxidation thermodynamics and processing history. This interfacial chemistry directly affects the relative stability of the orthorhombic, tetragonal, and monoclinic phases. A moderate and spatially controlled oxygen‐vacancy concentration can help stabilize the polar orthorhombic phase and suppress monoclinic‐phase formation, whereas excessive vacancy accumulation promotes leakage, local field enhancement, and eventual dielectric breakdown. Therefore, the improved cycling stability often observed with electrodes based on W or TiN should be understood as the result of controlled interfacial oxygen exchange, vacancy redistribution, suppression of the dead layer, and subsequent phase stabilization [17, 86, 87, 88, 89].

From a different structural perspective focusing on the atomic scale, Cheng et al. proposed a fatigue mechanism originating from a reversible transition between the polar orthorhombic (*Pbc2_1_*) and antipolar orthorhombic (*Pbca*) phases [63]. Using aberration‐corrected STEM combined with annular bright‐field imaging, they analyzed the oxygen atomic column shifts of each phase and showed that the fatigued film becomes dominated by the *Pbca* phase. As shown in Figure 3d–f, the alternating displacements of the off‐center oxygen columns along the [001] and its antiparallel direction, observed throughout the fatigued capacitor, are the characteristic feature of the antipolar *Pbca* phase. This atomic‐scale observation provides direct evidence for a *Pbc2_1_* to *Pbca* phase transition as the origin of polarization decay under prolonged low voltage cycling. Under low voltage cycling, the fatigued state could be restored to the polar *Pbc2_1_* phase by applying a higher voltage, indicating that the transition is reversible and occurs without permanent structural change or oxygen vacancy generation. In contrast, under high voltage cycling conditions, an interfacial non‐polar tetragonal (*P4_2_/nmc*) layer formed near the electrode and DFT calculations further revealed the existence of a critical effective field above which the polar *Pbc2_1_* phase is stabilized, whereas below this threshold the antipolar *Pbca* phase becomes energetically stable. Based on these results, the authors proposed that the depolarization field generated by this interfacial layer reduces the effective field within remaining polar region below the critical value, thereby driving the *Pbc2_1_* to *Pbca* phase transition. The partial recovery of the polarization under higher voltage further supports the conclusion that the same phase transition governs both regimes. These observations suggest that fatigue can arise from reversible polar to antipolar phase transition rather than from conventional defect generation or domain pinning mechanisms.

From a thermodynamic perspective, the phase evolution during long‐term electrical cycling can be understood as a relaxation process driven by free energy minimization. Continuous electrical cycling, accompanied by progressive defect generation, charge trapping, and oxygen vacancy accumulation, gradually increases structural disorder. As a result, the local free energy landscape is gradually modified, reducing the stability of metastable ferroelectric orthorhombic phase while favoring energetically more stable non‐polar phases, such as the monoclinic or antipolar orthorhombic phases [65]. This process can be interpreted as a gradual relaxation toward its thermodynamic ground state, in which the ferroelectric phase becomes destabilized over extended time scales [90].

Despite the strong support for defect‐ and phase‐driven interpretations, alternative mechanisms have also been proposed. Huang et al. reported that, in Y‐doped HfO_2_ thin films fabricated by pulsed laser deposition, fatigue is predominantly governed by domain‐wall pinning associated with carrier injection at shallow defect centers [91]. A key observation is that the activation energy of fatigue in this system is significantly lower than the migration barrier of oxygen vacancies, suggesting that oxygen vacancy diffusion is not the dominant mechanism. The strong dependence of fatigue rate on cycling field amplitude and frequency further supports this interpretation. Fatigue becomes most pronounced near the *E_c_*, where domain wall density is maximized, and at lower frequencies, where carrier migration and trapping processes are more effective. Moreover, fatigued devices were partially recovered after mild thermal annealing, suggesting that at least part of the fatigue originates from reversible defect states rather than irreversible structural degradation. This recovery can be understood as a thermally activated detrapping process. During cycling, charge carriers injected from the electrode under the applied high field become trapped at shallow defect sites near domain walls, where they electrostatically couple with the bound charges of the walls and locally impede polarization switching. Mild thermal annealing then provides sufficient thermal energy to release these carriers from the shallow traps, partially restoring domain wall mobility. The mild thermal treatment required for this recovery further suggests that permanent interfacial degradation, such as dead‐layer formation, is not always the dominant fatigue mechanism. These differing observations indicate that multiple mechanisms in HfO_2_‐based ferroelectrics can coexist and compete, with the dominant mechanism depending on dopant species, deposition method, and cycling conditions.

Building on the importance of cycling conditions, the dominant degradation pathway shifts as the operating field increases from near the *E_c_* to high overdrive states rather than acting as completely independent mechanisms. In practical devices, the programming field is often higher than *E_c_* to accelerate polarization switching. Such overdrive operation reduces switching time by enhancing nucleation and domain wall propagation, but it can also modify the dominant degradation pathway. A high density of nuclei can be generated almost simultaneously at grain boundaries, phase boundaries, and electrode interfaces, producing a rapidly evolving domain wall network with strong local field inhomogeneity [92, 93, 94]. In addition, high electric fields enhance carrier injection from electrodes, field‐assisted oxygen‐vacancy generation and migration, trap formation, and local leakage‐path formation. Under aggressive overdrive pulses, degradation should therefore be interpreted not only in terms of field amplitude but also in terms of pulse duration, duty cycle, local Joule heating, and the probability of time‐dependent dielectric breakdown. Even when high‐field pulses are short, the injected charge, local field concentration, and defect generation per operation can accelerate fatigue and dielectric degradation [46, 95, 96, 97]. Near *E_c_*, fatigue is more strongly associated with incomplete or unsaturated switching, domain‐wall pinning, and time‐dependent charge trapping, whereas high‐overdrive operation increasingly couples these processes to field‐enhanced defect generation and leakage. Accordingly, fatigue should be analyzed as a function of not only cycle number but also overdrive ratio, pulse width, duty cycle, waveform, and operating field [46, 91, 98].

In this context, fatigue can be viewed as a degradation of finite‐pulse switching kinetics. Generated oxygen vacancies, trapped charges, domain‐wall pinning centers, and cycling‐induced non‐polar or antipolar phases broaden the distribution of local switching barriers and reduce the fraction of domains that can switch within a fixed programming pulse. This kinetic degradation may appear as delayed or incomplete switching, broadened or split switching‐current peaks, positive/negative switching asymmetry, and increased cycle‐to‐cycle variability before a large decrease in quasi‐static *P_r_* becomes apparent. Therefore, correlating fatigue with switching dynamics is essential for practical devices, where a fixed pulse amplitude and width must reliably induce deterministic polarization reversal.

The dual role of defects is important in this context. A moderate density of residual impurities or grain‐boundary defects can locally lower the nucleation barrier and accelerate the median switching response, but the same spatially nonuniform defect landscape broadens the switching‐time distribution. During prolonged cycling, newly generated oxygen vacancies and trapped charges therefore do not simply shift the average switching time; they also increase the distribution width of local switching times and switching fields. As a result, some regions may still switch rapidly, whereas other regions fail to switch within the fixed programming pulse, producing incomplete *Q_sw_(t_p_)*, broadened or split switching‐current peaks, and stochastic write failure [61].

To comprehensively evaluate these complex degradation pathways, in addition to *P_r_*, *E_c_*, leakage current, and endurance cycles, imprint and dielectric loss should be considered as critical reliability metrics for HfO_2_‐based ferroelectric devices. Imprint reflects the development of a cycling‐induced internal bias field and is typically manifested as a horizontal shift or asymmetry of the P‐E hysteresis loop. It can be evaluated from the shift of the positive and negative coercive fields (*E_c_* ^+^ and *E_c_* ^−^, respectively), for example as *E_imp_* = (*E_c_* ^+^ + *E_c_* ^−^)/2 under a signed‐field convention. Microscopically, imprint is closely associated with asymmetric charge trapping, oxygen‐vacancy redistribution, and interfacial redox reactions [84]. Once an internal bias field is established, it superimposes on the externally applied bipolar field and produces unequal effective fields during the two switching half‐cycles, thereby promoting directional defect drift, incomplete switching, and accelerated fatigue [64, 84, 99, 100]. Dielectric loss provides another important diagnostic of reliability degradation. Depending on the measurement protocol, it can be evaluated from the small‐signal loss tangent, tan *δ*, or from the non‐switching energy‐loss component of large‐signal P‐E measurements. An increase in dielectric loss during cycling indicates that a larger fraction of the applied electrical energy is dissipated through leakage current, trap‐assisted conduction, interfacial dead‐layer formation, or incomplete polarization switching rather than reversible ferroelectric switching. Such non‐switching dissipation can accelerate Joule heating, defect generation, leakage‐path formation, and eventual dielectric breakdown [97, 101, 102, 103]. Therefore, systematic monitoring of imprint and dielectric loss is necessary for a complete assessment of fatigue and total device reliability.

Overall, fatigue in HfO_2_‐based ferroelectrics remains a major limitation for device performance, particularly in terms of endurance, which determines the number of reliable switching cycles. As conceptually summarized in Figure 3g, fatigue arises from the interplay among multiple microscopic mechanisms and their corresponding macroscopic effects. At the microscopic level, oxygen vacancy generation and redistribution, domain‐wall pinning by trapped carriers, interfacial instability at the electrode‐ferroelectric interface and structural phase degradation interact in a complex manner during repeated electrical cycling. These processes collectively lead to macroscopic degradation behaviors, including progressive polarization loss that reduces device reliability, and ultimately cycling‐induced dielectric breakdown. Because no single mechanism can fully explain fatigue across all device systems, and because the relative contribution of each mechanism depends strongly on factors such as dopant, deposition conditions, and electrical cycling parameters, improving endurance and suppressing polarization degradation require design strategies that simultaneously address these coupled degradation processes. Such controls can be achieved through fabrication optimization, defect engineering via doping and thermal treatment to control vacancy concentration and distribution [104, 105, 106, 107, 108]. In addition, careful selection of electrode materials plays an important role as it influences oxygen exchange and defect formation at the interface [21, 67, 88, 109, 110]. Thus, extensive research efforts have been devoted to reducing fatigue and enhancing endurance by combining materials and interface engineering strategies. In the following section, we discuss various approaches from a device perspective, including strategies for achieving wake‐up‐free or fast wake‐up behavior and methods for suppressing fatigue and improving endurance.

## 3 Wake‐Up‐Free and Fast Wake‐Up Design Strategies

Recently, significant efforts have been directed toward wake‐up‐free design strategies that suppress or eliminate the wake‐up phenomenon. In parallel, approaches that rapidly induce wake‐up during the initial cycling stage have also been proposed, particularly in cases where complete suppression of wake‐up is difficult. Such wake‐up‐free or fast wake‐up behavior directly contributes to improved device reliability and process simplification by enabling stable ferroelectric properties without extensive initial electrical conditioning or with only minimal initial cycling [48, 49, 111].

### 3.1 Doping and Composition

The wake‐up behavior in HfO_2_‐based ferroelectrics can be effectively controlled by the type and concentration of dopant elements. Numerous studies have investigated the mechanisms governing ferroelectricity in these materials through elemental doping and composition control [119]. Among various dopants, Zr is widely recognized as a representative dopant that forms a stable solid solution in HfO_2_ lattice due to its similar ionic radius and crystallographic compatibility with Hf. As a result, Zr plays a central role in stabilizing the ferroelectric orthorhombic phase [33, 120]. Based on this composition‐dependent phase stability, recent studies have systematically examined the influence of Zr content on wake‐up behavior during electrical cycling.

**FIGURE 4:**
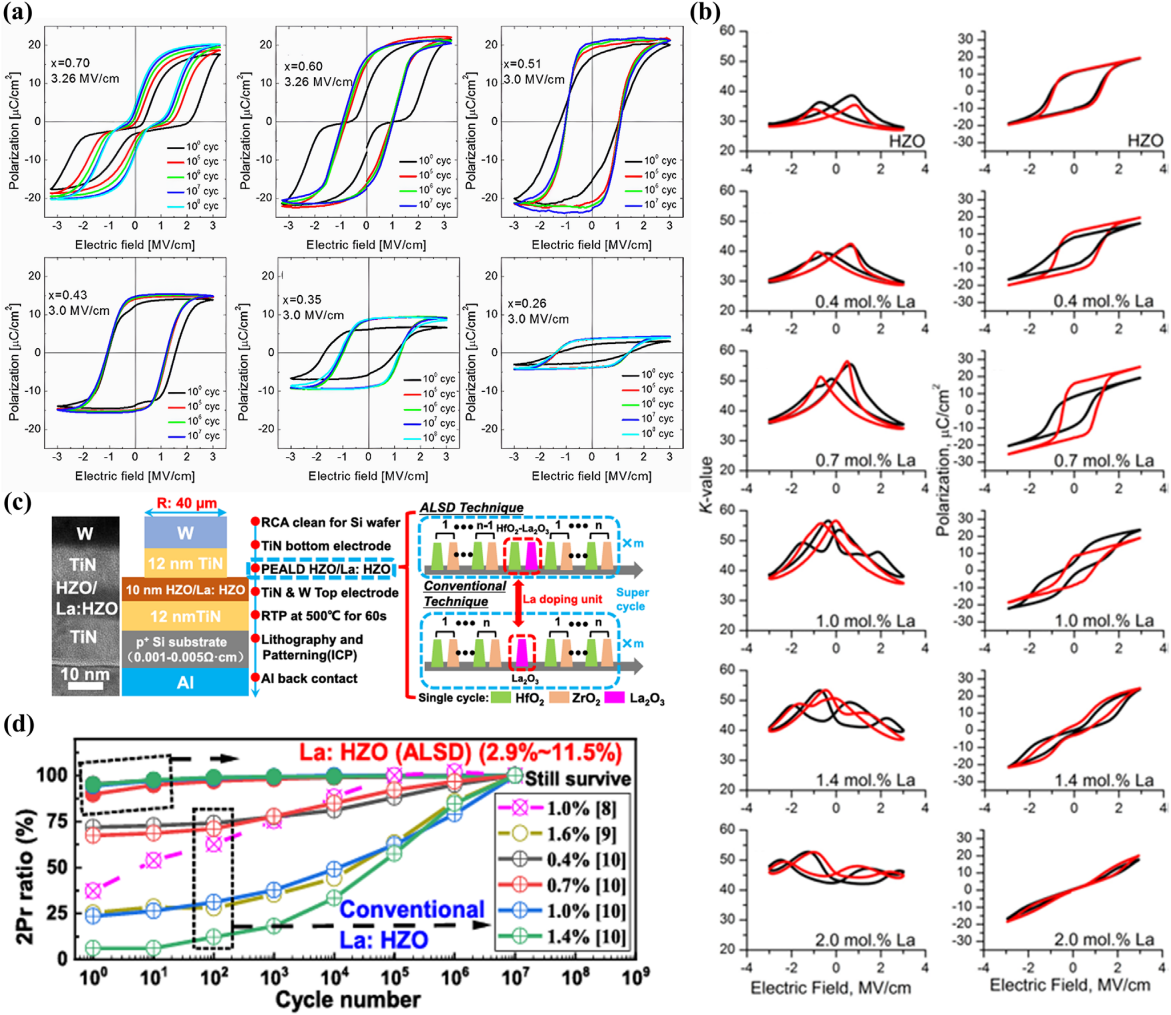
Strategy for wake‐up‐free through doping and process engineering. (a) Composition‐dependent wake‐up behavior of Hf_1‐_ *_x_* Zr*_x_* O_2_ thin films. Extracted P‐E hysteresis loops show distinct wake‐up characteristics depending on Zr contents, where depolarization effects are minimized near *x* = 0.43, leading to fast wake‐up behavior. Reproduced with permission [112]. Copyright 2016, American Chemical Society. (b) *k*‐E and P‐E hysteresis loops of HZO and HZLO‐based stacks with different La concentration. Black curves correspond to the pristine state, whereas red curves correspond to the measurements after wake‐up cycling of 10^5^ cycles. Reproduced with permission [44]. Copyright 2019, AIP Publishing LLC. (c) Process flow of the HZO/La:HZO MFM devices and illustration of ALSD and conventional doping methods for La:HZO deposition. (d) Electrical cycling results of La:MFM devices measured at 3 MV cm^−1^. Reproduced with permission [113]. Copyright 2022, IEEE.

Park et al. showed that the wake‐up behavior strongly depends on Zr content in Hf_1‐_ *_x_* Zr*_x_* O_2_ thin films, where the relative fractions of the initial tetragonal phase and the ferroelectric orthorhombic phase vary significantly with composition [112]. As shown in Figure 4a, the wake‐up behavior can be categorized into three regimes depending on Zr content (*x*). At low Zr contents (*x* = 0.26 and 0.354), films exhibit ferroelectric‐like behavior with reduced *P_r_*, whereas at high Zr contents (*x* = 0.6 and 0.7), highly distorted P–E hysteresis loops are observed. In contrast, intermediate compositions (*x* = 0.43 and 0.51) show well‐defined ferroelectric behavior with distinct wake‐up characteristics. In particular, even within this intermediate regime, the extent of wake‐up differs significantly. The film with *x* = 0.51 shows a pronounced increase in the slope of the P‐E hysteresis loops near the *E_c_* during cycling, along with a large variation in *P_r_*, whereas the film with *x* = 0.43 shows negligible change. This behavior can be understood in terms of the interplay between phase distribution and defect‐induced interfacial effects. At compositions deviating from the optimal range, a larger fraction of the tetragonal phase is stabilized, particularly near electrode interfaces where oxygen vacancies tend to accumulate. These regions act as non‐ferroelectric interfacial layers, generating depolarization fields that suppress the initial polarization and require substantial defect redistribution during cycling, resulting in pronounced wake‐up behavior. In contrast, at compositions near *x*∼0.5, the stabilization of the tetragonal phase is reduced, which limits vacancy accumulation at the interfaces. As a result, the interfacial non‐ferroelectric layer becomes thinner, reducing the depolarization field and minimizing the difference between the pristine and cycled states. Consequently, at optimized Zr compositions, wake‐up is either strongly suppressed or proceeds rapidly enabling fast or quasi wake‐up‐free operation. Consistently, Mittmann et al. reported that a Zr content of approximately 50% provides the most stable electrical cycling behavior [33].

In addition to conventional compositional optimization approaches, an alternative strategy based on cation‐excess engineering has recently been proposed. Wang et al. demonstrated that Hf(Zr)_1+_ *_x_* O_2_ thin films with excess cations can spontaneously stabilize a polar rhombohedral phase through intercalation of additional Hf/Zr atoms into the lattice [31]. The intercalated cations expand the lattice and generate internal stress, which stabilizes the rhombohedral structure and its intrinsic ferroelectric nature. As a result, the material can exhibit ferroelectric behavior directly from the pristine state without requiring defect‐mediated phase transitions, thereby suppressing or even eliminating wake‐up effect. This approach suggests that compositional engineering not only influences phase distribution but can also fundamentally modify the polarization formation mechanism itself, thereby enabling wake‐up‐free behavior.

Beyond Zr doping, various elemental doping strategies have been explored to control the wake‐up behavior in HfO_2_‐based ferroelectrics. Among these, La doping has been widely reported to influence both the thermodynamic stability and kinetic behavior of oxygen vacancies, thereby tuning their concentration and charge state. In particular, La incorporation shifts the Fermi level and modifies defect energetics, enabling a more controlled defect distribution while also affecting phase stability and the formation of the ferroelectric orthorhombic phase [114]. Kozodaev et al. systematically investigated the effects of low‐concentration La doping (0.4–2.0 mol%) in HZO‐based metal‐ferroelectric‐metal (MFM) capacitors [44]. Their results showed that low La doping (0.4 and 0.7 mol%) improves crystal stability and endurance by suppressing monoclinic phase formation and reducing domain pinning. In addition, leakage current is reduced and oxygen vacancy accumulation is limited, thereby enabling more homogeneous polarization switching during electrical cycling. However, despite these improvements, wake‐up behavior is not completely eliminated as shown in Figure 4b. Instead, the films exhibit limited polarization in the pristine state, followed by a reactivation of polarization during cycling, indicating that the wake‐up behavior is governed by La doping. These observations indicate that doping enables a controlled wake‐up process with reduced amplitude, achieving a balance between improved endurance and stable ferroelectric performance rather than a fully wake‐up‐free implementation. In addition to single element doping, co‐doping strategies have been explored to further tune defect and phase behavior. Popovici et al. analyzed the effects of co‐doping with heterogeneous elements, such as (La, Y) and (La, Gd) on the electrical cycling characteristics of HZO thin films [108]. Lomenzo et al. further demonstrated that Al and Si co‐doping significantly influence wake‐up behavior and ferroelectric properties by modifying phase distribution and defect characteristics in HfO_2_‐based thin films [118].

Beyond the individual effects of specific dopants, a general correlation can be established between dopant chemistry, oxygen vacancy behavior, and phase evolution in HfO_2_‐based ferroelectrics. Aliovalent doping modifies both the concentration and spatial distribution of oxygen vacancies, thereby altering the local defect landscape, initial electric field, and relative phase stability. During electrical cycling, the redistribution of these defects and the associated relaxation of internal fields drive structural transformations toward the ferroelectric state, giving rise to the wake‐up effect. This behavior is consistently observed across various dopant systems, indicating that wake‐up is fundamentally governed by defect‐mediated phase evolution and the coupling between defect dynamics and phase stability. To provide a clearer overview of this dopant‐dependent relationship, Table 1 summarizes the representative effects of various doping strategies on defect/structure‐related mechanisms, phase stability, and wake‐up behavior. This comparison highlights that different dopants influence wake‐up characteristics through distinct but closely coupled pathways involving defect redistribution, internal‐field modulation, and phase stabilization.

**TABLE 1:** Summary of dopant‐dependent defect and structure‐related mechanisms, phase stability and wake‐up behavior in HfO_2_‐based ferroelectrics.

Dopant / Strategy	Defect/structure‐related mechanism	Effect on phase stability	Wake‐up behavior	References
Zr (∼50%)	Suppression of interfacial oxygen vacancy accumulation and depolarization field	o‐phase stabilization near optimized composition	Fast / weak wake‐up	[112]
La	Modification of oxygen vacancy energetics, charge state, and Fermi level / leakage current reduction	Reduction of domain pinning and suppression of m‐phase	Controlled / reduced wake‐up	[44, 57, 114]
Y	Leakage current improvement, switching field and internal‐field‐induced domain pinning	Suppression of m‐phase	Depinning‐driven wake‐up	[115]
Al	Passivation of oxygen vacancy induced defect states and suppression of excessive oxygen vacancy generation	Tuning o/t phase balance and can suppress m‐phase	Composition/distribution‐dependent; nearly wake‐up‐free behavior under optimized conditions	[116, 117]
Si	Formation of defect/interface‐related built‐in fields and promotion of charged‐defect redistribution during cycling	Stabilization of t‐rich phase and field‐induced reversible t to o transition	Pinched or antiferroelectric‐like hysteresis and defect redistribution‐driven wake‐up	[43, 118]
La‐Y / La‐Gd co‐doping	Lattice distortion and intrinsic stress generation through ionic‐radius mismatch; modulation of oxygen vacancy related internal bias and domain pinning	Suppression of m‐phase and enhancement of t/o phase fractions	Controlled but occasionally prolonged wake‐up	[108]
Cation intercalation	Lattice expansion and internal stress generation	Stabilization of r‐phase directly	Wake‐up‐free / weak wake‐up	[31]

### 3.2 Process Engineering

Wake‐up‐free cases have also been reported in HfO_2_‐based ferroelectric thin films through process optimization and the introduction of advanced deposition techniques that suppress or eliminate the wake‐up phenomenon. Weng et al. investigated wake‐up‐free characteristics in La‐doped HZO (HZLO) thin films fabricated using atomic layer‐specific doping (ALSD) [113]. In this process, as shown in Figure 4c, La dopants introduced via plasma‐enhanced atomic layer deposition (PEALD) are selectively incorporated at specific atomic layers, which effectively stabilizes the orthorhombic phase compared with conventional doping deposition approaches. This selective incorporation of dopants enables precise control over the local chemical environment and minimizes the formation of non‐ferroelectric regions associated with unfavorable dopant configurations. As a result, a large fraction of the ferroelectric phase is already established in the pristine state, reducing the need for defect redistribution or phase transformation during electrical cycling. Consequently, the typical wake‐up behavior, which originates from defect‐mediated phase evolution, is effectively suppressed. As a result, positive‐up negative‐down (PUND) measurements exhibit excellent initial switching characteristics with negligible wake‐up behavior and high *P_r_* from the pristine state, suggesting the feasibility of process‐based wake‐up‐free or fast wake‐up behavior, as shown in Figure 4d. This demonstrates that wake‐up can be fundamentally avoided by initializing the system with an optimized phase distribution through controlled doping strategies. Similarly, Chou et al. realized wake‐up‐free characteristics by controlling crystallization behavior and defect distribution in HZO thin films using an atomic layer crystallization induced by substrate bias (ALCISB) technique, in which a substrate bias is applied during the ALD process [121]. The ALCISB approach enhances atomic mobility during film growth, promoting the formation of the ferroelectric orthorhombic phase while simultaneously reducing oxygen vacancy concentration. Consequently, stable ferroelectric properties with high *P_r_* and excellent endurance are obtained without requiring an additional electrical wake‐up process.

### 3.3 Frequency and Time Domain Control of Switching‐Kinetics

**FIGURE 5:**
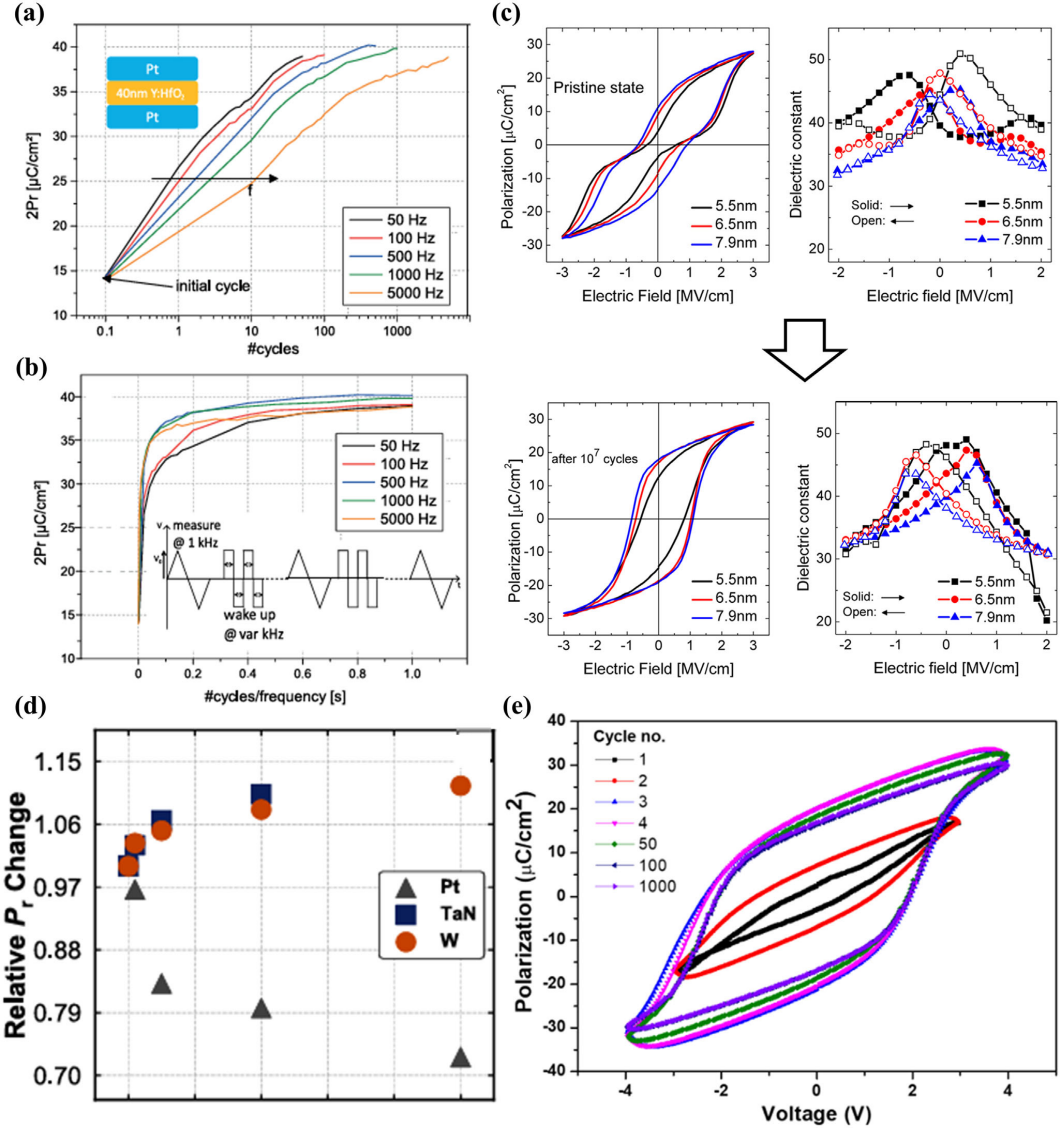
Strategy for wake‐up‐free through frequency control, thickness tuning, electrode selection, and epitaxial strain. (a) Double remanent polarization (2*P_r_*) as a function of the number of cycles at different frequencies. Higher wake‐up frequencies require a larger number of cycles to reach the same polarization level. (b) 2*P_r_* as a function of the duration of the applied electrical field (number of cycles/frequency). The inset shows the cycling and measurement sequence. Hysteresis is measured at 1 kHz, while the wake‐up cycle frequency is varied from 50 Hz to 5 kHz at 3.25 MV cm^−1^. Reproduced with permission [68]. Copyright 2016, AIP Publishing LLC. (c) P‐E and *k*‐E curves of Hf_0.5_Zr_0.5_O_2_ films with thicknesses of 5.5, 6.5, and 7.9 nm for pristine state and after wake‐up. Reproduced with permission [51]. Copyright 2015, AIP Publishing LLC. (d) Relative *P_r_* as a function of the number of cycles for symmetric MIM structures with Pt (gray triangles), TaN (blue squares), and W (orange circles) electrodes. Reproduced with permission [73]. Copyright 2020, American Chemical Society. (e) P‐V hysteresis loops measured for an LSMO/HZO/LSMO capacitor with a 9 nm HZO film measured at 1 kHz. The *P_r_* remains nearly unchanged from the fourth to the 10^3^rd cycle, indicating negligible wake‐up behavior. Reproduced with permission [30]. Copyright 2018, Springer Nature Limited.

Electrical driving conditions, particularly the applied voltage frequency and the effective duration of electric field application, serve as key design parameters for achieving fast wake‐up behavior. Starschich et al. investigated the relationship between wake‐up behavior and cycling frequency in 40 nm thick Y‐doped HfO_2_ films and reported that a large number of cycles is required to reach the same *P_r_* as the frequency increases as shown in Figure 5a [68]. This finding indicates that wake‐up behavior is governed primarily by the total effective duration of electric field application rather than by the number of cycles itself. Consistently, similar wake‐up characteristics were observed when the total electric field application time was kept constant under different frequency conditions as shown in Figure 5b. These results suggest that the wake‐up process is dominated by ion diffusion related mechanisms, such as electric field driven migration and redistribution of oxygen vacancies, rather than by domain switching. This behavior further indicates that the wake‐up process is highly sensitive to defect redistribution governed by ion mobility. This is further supported by the pronounced acceleration of wake‐up at elevated temperatures, demonstrating that thermally activated oxygen vacancy migration enhances the defect redistribution and thereby facilitates faster stabilization of the ferroelectric state. Consequently, fast or quasi fast wake‐up behavior can be attributed to the rapid equilibration of the defect distribution under conditions of enhanced vacancy mobility, enabling quicker stabilization of the ferroelectric response. Li et al. further investigated the frequency‐dependent wake‐up behavior in Si‐doped HfO_2_ thin films and found that increasing cycling frequency and decreasing cycling electric field amplitude lead to similar effects [98]. Specifically, both conditions result in increased distortion of saturated P‐E hysteresis loops and more pronounced splitting of the switching current peaks. In addition, frequency‐ and temperature‐dependent harmonic analyses performed on Sr‐doped HfO_2_ revealed that wake‐up and fatigue behaviors can be described by activation energies with distinct frequency dependencies [52]. These results indicate that fast wake‐up should be evaluated not only by the number of cycles required to reach a saturated *P_r_*, but also by how rapidly the switching‐field and switching‐time distributions stabilize under application‐relevant pulse conditions. Thus, frequency, pulse width, and waveform engineering provide operation‐level routes for controlling defect redistribution and finite‐pulse switching kinetics simultaneously.

In addition to pulse conditions, the initial defect and microstructural landscape set by processing and electrode materials also determines the switching‐time distribution. Residual impurities introduced during ALD can reduce the median switching time while increasing the distribution width [61], whereas electrode‐dependent phase stability and grain size can change the NLS width parameter and low‐voltage switched fraction [122]. Therefore, frequency, pulse width, waveform, deposition process, and electrode/interface design should be treated as coupled knobs for engineering both the average switching speed and the statistical distribution of switching events.

### 3.4 Others

Furthermore, numerous studies have sought to identify key factors influencing wake‐up behavior and to achieve fast wake‐up characteristics. Park et al. systematically investigated thickness dependent wake‐up behavior in ultrathin HZO films with thicknesses below 8 nm as shown in Figure 5c [51]. In the pristine state, reducing film thickness decreases the relative surface energy of the tetragonal phase, leading to a more pronounced antiferroelectric‐like response. After electrical cycling, ferroelectric properties were recovered across all thicknesses. However, thinner films exhibited a reduction in the coercive voltage (*V_c_*), which was attributed to enhanced depolarization effects associated with thickness scaling. This behavior can be understood in terms of the thickness‐dependent phase stability and depolarization‐driven internal field evolution. In ultrathin films, the relative surface energy favors non‐ferroelectric phases in the pristine state, leading to an initially suppressed polarization response. However, during electrical cycling, the phase transition toward the ferroelectric phase proceeds rapidly because the transformation is thermally activated with a relatively low activation energy. In addition, the enhanced depolarization effect in thinner films reduces the effective *E_c_*, facilitating easier phase transformation and polarization switching. These results suggest that initial non‐rigid behavior can be intentionally introduced through thickness control and subsequently converted into fast wake‐up behavior during electrical cycling.

In another study, the influence of electrode materials on wake‐up and fatigue behavior in metal‐insulator‐metal (MIM) structures was analyzed. Fields et al. compared HZO capacitors with Pt, TaN, and W electrodes and showed that devices with noble metal electrodes, e.g. Pt, which effectively block oxygen ion transport, undergo a phase transition from the ferroelectric orthorhombic phase to the non‐ferroelectric monoclinic phase during electrical cycling, resulting in dominant fatigue behavior [73]. In contrast, when nitride‐based electrodes, e.g. TaN, which can absorb oxygen and oxygen vacancies, or reactive metal electrodes, e.g. W, are used, defect redistribution and phase transition from the tetragonal to the orthorhombic phase are promoted, leading to enhanced wake‐up behavior, as presented in Figure 5d. These results indicate that the interfacial chemistry between the electrode and HZO layer plays a critical role in determining phase evolution and defect dynamics during electrical cycling. Noble metal electrodes such as Pt block oxygen transport, limiting oxygen exchange at the ferroelectric/metal interface. Consequently, oxygen vacancies are mainly redistributed within the HZO layer, promoting relaxation of the ferroelectric orthorhombic phase toward the thermodynamically stable monoclinic phase and leading to fatigue behavior. In contrast, nitride electrodes such as TaN can absorb oxygen and oxygen vacancies, enabling defect exchange across the interface and modifying the overall defect concentration in HZO. For W electrodes, partial oxidation near the interface is thermodynamically possible because of the low formation energy of tungsten oxides. The resulting tungsten oxide interfacial phases may act as sinks or sources of oxygen vacancies, similarly to TaN electrodes, facilitating defect redistribution and field‐induced tetragonal‐to‐orthorhombic phase transformation. Therefore, wake‐up behavior is strongly influenced not only by bulk HZO properties but also by interfacial electrode chemistry and oxygen vacancy exchange.

Strain engineering in epitaxially grown HfO_2_‐based films has also emerged as an effective approach for suppressing or eliminating the wake‐up behavior. Song et al. demonstrated that epitaxial La‐doped Hf_0.5_Zr_0.5_O_2_ thin films exhibit a significantly suppressed wake‐up behavior compared to polycrystalline counterparts [54]. They attributed this behavior to stabilization of the ferroelectric phase through epitaxial strain and improved structural uniformity, which minimizes defect redistribution during cycling and enables nearly wake‐up‐free behavior from the pristine state.

More recently, several studies have reported the use of epitaxial strain to stabilize the rhombohedral phase. Petraru et al. demonstrated that epitaxial HfO_2_‐based thin films stabilized in the rhombohedral phase via substrate‐induced strain exhibit clear ferroelectric switching directly in the as‐grown state without requiring wake‐up cycling [71]. This behavior originates from direct stabilization of the polar rhombohedral phase under epitaxial constraints, which reduces the need for defect redistribution or electric field‐induced phase transformation typically required in polycrystalline orthorhombic systems. Similarly, Wei et al. reported that epitaxial strain can stabilize the rhombohedral phase and enable wake‐up‐free ferroelectric behavior as shown in Figure 5e [30, 70]. Through detailed structural characterization and theoretical calculations, they demonstrated that the observed wake‐up‐free behavior originates from the direct stabilization of a polar rhombohedral phase under epitaxial compressive strain. Comparable wake‐up‐free behavior has also been observed in ferroelectric rhombohedral ZrO_2_ thin films, in which epitaxial compressive strain directly stabilizes the polar phase in the as‐grown state [123]. Considering the strong chemical similarity between HfO_2_ and ZrO_2_, these findings provide additional evidence supporting the generality of strain‐induced rhombohedral ferroelectricity.

Recent studies have further demonstrated that wake‐up‐free behavior can be achieved through interface and seed‐layer engineering. In particular, TiO_2_ seed layers and ultrathin dielectric interlayers such as HfO_2_, ZrO_2_, and Al_2_O_3_ were shown to suppress interfacial oxygen‐vacancy generation and domain pinning, thereby stabilizing the ferroelectric orthorhombic phase from the pristine state and simultaneously improving endurance reliability [124, 125]. Furthermore, several studies have examined the influence of process‐ and driving‐related parameters, including operating temperature [46, 126] and ozone dose control [111] during the ALD process, on the wake‐up behavior of HfO_2_‐based thin films.

## 4 Strategies for Enhancing Endurance of HfO_2_‐Based Ferroelectrics

In line with the mechanism‐driven framework established above, the engineering strategies discussed in this section are organized according to the reliability mechanisms that they primarily address. Doping and composition engineering are considered in relation to oxygen‐vacancy formation energy, defect distribution, and polymorphic phase stability. Electrode, interlayer, and interface engineering are discussed in terms of interfacial oxygen chemical potential, redox reactions, charge trapping, and dead‐layer suppression. Process engineering and multilayer/nanolaminate designs are treated as approaches for controlling stoichiometry, defect transport, stress distribution, and phase stabilization. Operation‐level parameters, including waveform, pulse duration, frequency, and voltage‐step protocols, are then revisited in the Summary and Outlook as methods for controlling defect kinetics and charge injection during cycling. As discussed in the previous sections, prolonged cycling promotes the generation of additional oxygen vacancies and charge trapping, which gradually degrades polarization and ultimately leads to premature dielectric breakdown during continuous operation. Furthermore, HfO_2_‐based ferroelectrics possess relatively high *E_c_* (∼ 1.5 MV cm^−1^) compared to conventional perovskite ferroelectrics. This results in high operating voltages for polarization switching and imposes substantial electrical stress during cycling, thereby making dielectric breakdown a principal limiting factor for device endurance [39, 64, 127, 128]. When these factors act together, the high switching field accelerates defect‐driven degradation, reducing device lifetime to levels well below those required for practical applications. Consequently, various approaches have been explored to suppress defect‐driven degradation and reduce electrical stress during cycling. In this section, we discuss these strategies to address these challenges.

### 4.1 Electrode and Doping Engineering

**FIGURE 6:**
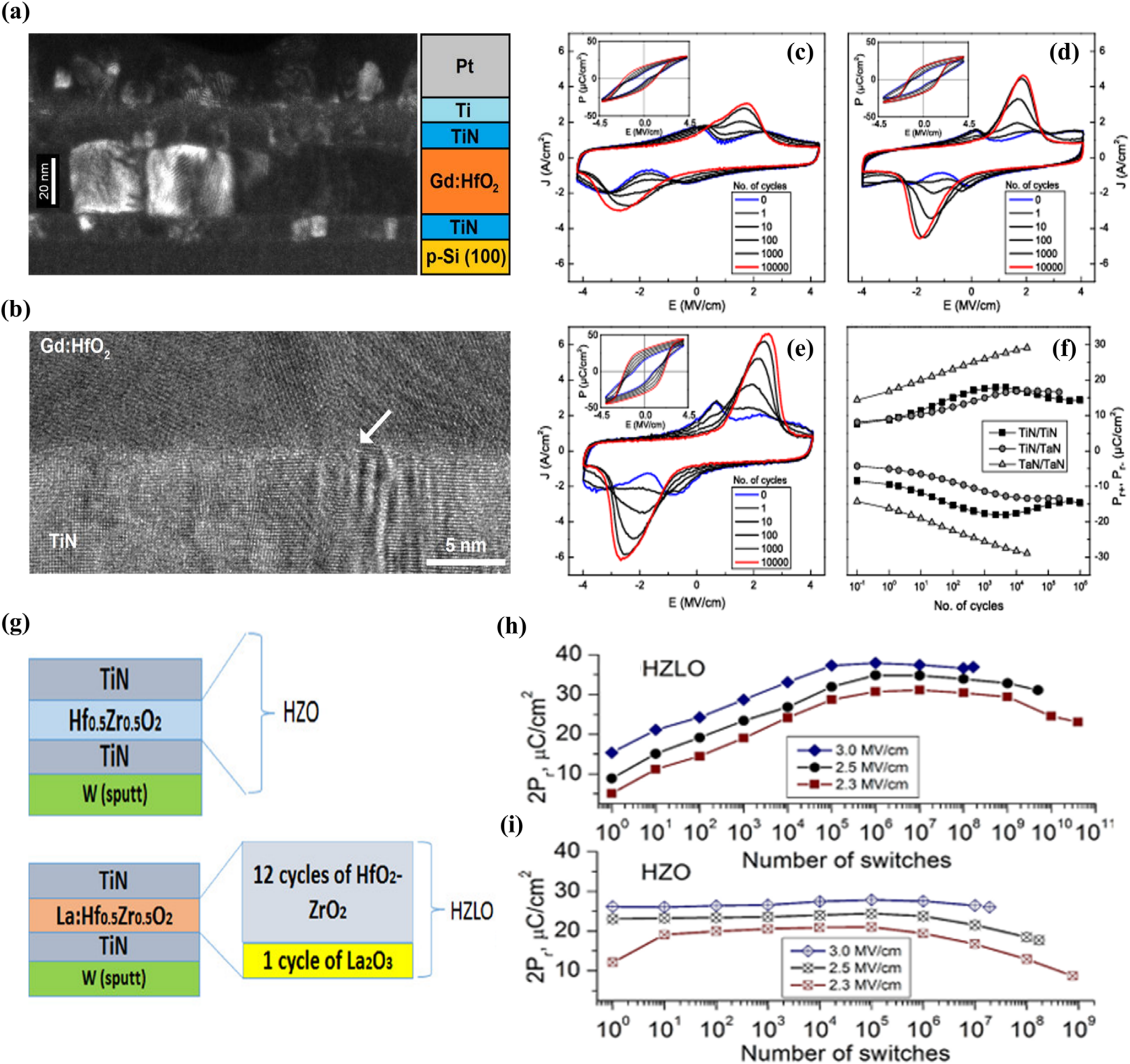
Cycling‐dependent ferroelectric behavior of HfO_2_‐based capacitors with different electrode configurations and doping strategies. (a) Cross sectional TEM image and corresponding schematic of the TiN/Gd:HfO_2_/TiN ferroelectric capacitor structure. (b) High‐resolution TEM image of the Gd:HfO_2_/TiN interface, highlighting the grain boundary region. (c–e) Wake‐up behavior of 10 nm Gd:HfO_2_ capacitors with (c) TiN/TaN, (d) TiN/TiN, and (e) TaN/TaN electrodes, showing J–E curves as a function of electrical cycling. Insets show corresponding P‐E hysteresis loops. (f) *P_r_* as a function of applied field cycles (4 MV cm^−1^) for all three electrode configurations. Reproduced with permission [21]. Copyright 2015, AIP Publishing LLC. (g) Schematic illustration of HZO and La_2_O_3_‐inserted HZLO capacitor structures. (h) Endurance characteristics of the HZLO capacitor under different electric fields. (i) Endurance characteristics of the HZO capacitor for comparison. Reproduced with permission [57]. Copyright 2017, American Chemical Society.

Since fatigue and endurance depend on the electrode‐ferroelectric interface, numerous studies have examined polarization cycling behavior using various upper and lower electrode configurations [22, 67, 109, 129, 130, 131]. Accordingly, electrode and interlayer engineering should be designed to control interfacial oxygen exchange rather than merely to select chemically inert contacts. Properly tuned reactive electrodes or ultrathin oxygen‐reservoir interlayers can stabilize the polar phase by regulating vacancy redistribution, whereas excessive interfacial reduction can increase leakage and accelerate breakdown [132, 133]. Hoffmann et al. systematically compared the cycling‐dependent ferroelectric behavior of Gd:HfO_2_‐based capacitors with different electrode combinations, as summarized in Figure 6a–f [21]. The structural features of the device are shown in Figure 6a,b. Cross sectional TEM images reveal a TiN/Gd:HfO_2_/TiN stack and the microstructure near the electrode interface, including grain boundaries, which can influence defect redistribution and dynamics during electrical cycling, as discussed in the previous sections.

Figure 6c–e presents the evolution of switching current density‐electric field (J‐E) and P‐E hysteresis loops under repeated electrical cycling for three electrode configurations. The asymmetric TiN/TaN configuration suppresses fatigue and maintains stable polarization until dielectric breakdown without a distinct fatigue regime. In contrast, the TiN/TiN configuration exhibits initial wake‐up behavior up to approximately 10^4^ cycles, followed by gradual contraction of the hysteresis loop, indicating the onset of fatigue prior to breakdown. The TaN/TaN configuration shows pronounced polarization enhancement during cycling without a clear fatigue regime, but experiences earlier dielectric breakdown, as shown in Figure 6f. These differences originate from the distinct interfacial oxidation behavior of the two nitride electrodes. TaN has a stronger tendency to form an oxynitride interfacial layer than TiN, extracting oxygen from the ferroelectric layer and generating a higher concentration of oxygen vacancies. The increased vacancy concentration stabilizes the polar orthorhombic phase and suppresses monoclinic phase formation, thereby reducing fatigue. However, the same vacancies also facilitate dielectric breakdown in HfO_2_ and lead to the earlier dielectric failure of capacitors with TaN electrodes despite their improved fatigue resistance. These results highlight that the trade‐off between fatigue resistance and dielectric lifetime in HfO_2_‐based capacitors is mediated by the oxygen vacancy concentration introduced through electrode oxidation, identifying interfacial chemistry as a critical design parameter for achieving high endurance.

Building on these observations, Alcala et al. extended the electrode comparison to nitride electrodes (TiN, TiAlN, and NbN), a metal electrode (W), and oxide electrodes (MoO_2_, RuO_2_, and IrO_2_) [134]. Nitride electrodes form interfacial oxide layers of 0.9–1.7 nm due to interfacial reactions, which increase oxygen vacancy concentration in the HZO layer by 0.6–0.8%. The resulting interface acts as a source of defects that propagate into the HZO film during cycling, contributing to gradual degradation. In contrast, oxide electrodes do not form detectable interfacial layers and maintain oxygen vacancy concentrations below 0.1%. However, they promote stabilization of the monoclinic phase and introduce large hysteresis shifts, which reduce ferroelectric response. These behaviors originate from the electrode oxidation thermodynamics. A stronger driving force for oxidation generates thicker interfacial reaction layers, which in turn increase oxygen vacancy concentration in HZO, thereby influencing the crystalline phase distribution. Because crystallization proceeds through an initial tetragonal phase, the local oxygen environment determines whether the subsequent transition leads to the orthorhombic or monoclinic phase. Accordingly, vacancy‐rich conditions favor polar orthorhombic phase while suppressing monoclinic phase.

The degradation pathway during cycling also depends strongly on interfacial chemistry. In nitrides, the pre‐existing reaction layer continuously introduces defects into the HZO film during cycling, eventually forming conductive paths that trigger dielectric breakdown. In contrast, W initially forms a conductive tungsten oxide layer that does not act as an insulating interfacial barrier. However, repeated cycling progressively oxidizes the tungsten oxide further, continuously increasing oxygen vacancy concentration in HZO until breakdown occurs. These results indicate that interfacial reactivity dominates oxygen vacancy concentration, phase fraction, and device reliability in HfO_2_‐based ferroelectrics. Overall, fatigue and endurance in HfO_2_‐based ferroelectrics are largely governed by how each electrode interacts with oxygen vacancy at the ferroelectric interface. Oxygen vacancies generated through electrode‐ferroelectric reactions can simultaneously stabilize the polar phase and suppress fatigue while also accelerating dielectric breakdown. Conversely, electrodes with low oxygen reactivity suppress oxygen vacancy generation but may be insufficient to stabilize the polar phase, often leading to increased monoclinic fraction and reduced ferroelectric response. Therefore, achieving high endurance requires deliberate control of electrode‐oxygen reactivity, either through electrode composition engineering or engineered interfacial layers to maintain an optimized vacancy concentration that stabilizes the polar phase while suppressing both fatigue and dielectric breakdown.

Beyond electrode engineering, chemical doping has also emerged as an effective approach for improving endurance in HfO_2_‐based ferroelectrics [41, 44, 98]. Chernikova et al. demonstrated that La doping in HZO significantly enhances endurance, as shown in Figure 6g–i [57]. As illustrated in Figure 6g, the HZLO structure is realized by periodically inserting La_2_O_3_ cycles into the HZO matrix during ALD growth. The endurance behavior of the La‐doped capacitor in Figure 6h shows a markedly improved polarization stability compared to the undoped HZO in Figure 6i, which exhibits earlier degradation. This improvement originates from two main effects. First, the *E_c_* is reduced by approximately 30%, enabling switching at lower applied electric fields and thereby suppressing premature dielectric breakdown. Second, La doping decreases leakage current by approximately three orders of magnitude, further lowering the probability of breakdown during repeated cycling. From a defect perspective, La^3+^ acts as an acceptor that shifts the Fermi level toward the midgap, thereby reducing leakage current. Although La incorporation increases oxygen vacancy concentration near the interface and results in a prolonged wake‐up stage, this effect occupies only a limited fraction of the total cycling lifetime. Consequently, the reduction in *E_c_* and leakage current dominates, leading to a significant improvement in endurance.

### 4.2 Additional Layer and Fabrication Process Engineering

**FIGURE 7:**
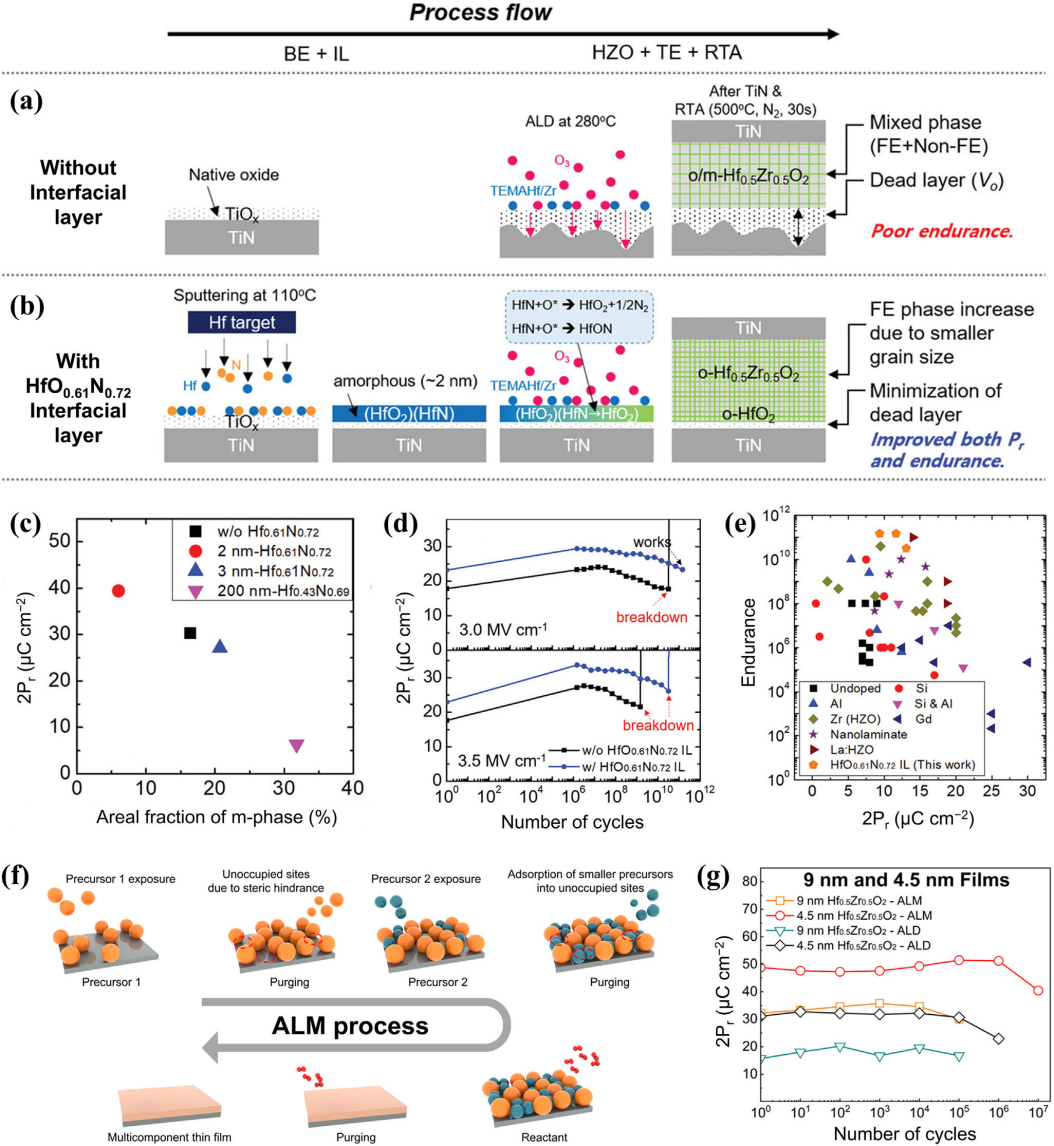
Endurance enhancement in HZO ferroelectric capacitors through interfacial layer insertion and ALM process. (a) Schematic of the MFM structure without an interfacial layer. (b) MFM structure with HfO_0.61_N_0.72_ interfacial layer. (c) 2*P_r_* of the HZO as a function of the areal fraction of monoclinic(m‐) phase with different thickness of HfO*_x_* N*_y_*. (d) Polarization evolution of 2 nm‐thick HfO_0.61_N_0.72_ inserted HZO during cycling at 3.0 MV cm^−1^, 3.5 MV cm^−1^. (e) Comparison of *P_r_* and endurance between 2 nm‐thick HfO_0.61_N_0.72_ inserted HZO and other reported HfO_2_‐based ferroelectric systems. Reproduced with permission [135]. Copyright 2021, Wiley‐VCH GmbH. (f) Schematic of the ALM process. (g) Cycling endurance and polarization retention of 9 and 4.5 nm ALD‐ and ALM‐grown HZO films. Reproduced with permission [136]. Copyright 2025, American Chemical Society.

In HfO_2_‐based capacitors, direct contact between the electrode and the ferroelectric layer can induce unfavorable interfacial reactions during deposition and annealing, leading to the formation of unintended interfacial dielectric layer and a reduction in the ferroelectric phase fraction [67, 137, 138]. Such interfacial degradation can promote polarization loss during repeated cycling and result in poor endurance [46]. To address these issues and improve cycling reliability, the insertion of additional layers either at the electrode‐ferroelectric interface or as a separate sublayer adjacent to the ferroelectric film has emerged as an effective strategy [12, 139, 140, 141]. Such additional layers can modify the interfacial defect chemistry, introduce internal interfaces that influence defect transport during cycling, or stabilize the ferroelectric phase, all of which may contribute to long‐term reliability. For example, Kim et al. demonstrated that inserting an HfO_0.61_N_0.72_ interfacial layer between the TiN bottom electrode and the HZO film can suppress interfacial degradation, reduce dead‐layer formation, and increase the ferroelectric phase fraction [135]. As illustrated in Figure 7a,b, the capacitor without the interfacial layer formed a mixed‐phase region and a defective dead layer after thermal processing, whereas structures with the interfacial layer exhibited a larger fraction of ferroelectric‐phase and a minimized dead layer. Consistent with this structural evolution, Figure 7c shows that the *P_r_* increases as the monoclinic phase fraction decreases, with the optimized 2 nm‐thick HfO_0.61_N_0.72_ interfacial layer producing the lowest monoclinic phase content and the highest 2*P_r_*. Notably, the enhancement was dependent on interlayer thickness, with a 2 nm‐thick HfO_0.61_N_0.72_ layer providing the highest polarization, whereas thicker interlayers resulted in lower 2*P_r_* and a larger monoclinic phase fraction. This interfacial modification reduced the rate of polarization degradation during cycling and extended endurance by suppressing premature breakdown, as shown in Figure 7d. The improvement is attributed to two coupled roles of the nitrogen‐containing interfacial layer. First, the interfacial layer acts as an oxygen diffusion barrier between the TiN electrode and the HZO film, suppressing the interfacial oxidation of the TiN electrode. This suppression reduces the formation of non‐ferroelectric interfacial phases, such as TiO_2_ and Ti‐O‐N materials, as well as the accompanying oxygen vacancies that contribute to subsequent dead‐layer formation and degradation. Second, the interfacial layer remains amorphous during HZO deposition and preserves the amorphous as‐deposited state of the HZO film, promoting more homogeneous crystallization during the subsequent annealing process. The resulting smaller and more uniform grains stabilize the polar orthorhombic phase through increased surface energy contribution. The beneficial effect was strongly thickness dependent, however, because thick HfO_0.61_N_0.72_ layers can transform into non‐ferroelectric monoclinic HZO after oxidation and annealing with reduced 2*P_r_*. Consequently, the 2‐nm thick interfacial layer provided the optimal balance between interfacial protection and ferroelectric phase stabilization, resulting in both the highest 2*P_r_* and the best endurance performance compared with both thicker interlayers and structures without an interfacial layer. These coupled effects indicate that interlayer engineering and optimizing the thickness of interlayer can simultaneously control the interfacial defect chemistry and ferroelectric phase stability of HfO_2_‐based ferroelectrics. Furthermore, as shown in Figure 7e, this interlayer‐engineered capacitor achieved a favorable balance between *P_r_* and endurance compared to previously reported HfO_2_‐based ferroelectric capacitors. These results demonstrate that interlayer engineering provides a practical approach for enhancing endurance by controlling interfacial defect processes that contribute to leakage‐path formation and dielectric breakdown.

Endurance can also be improved by inserting interlayers based on materials other than HfO_2_‐related compounds, with thin Al_2_O_3_ layers as a representative example [139, 142, 143, 144]. When placed at the electrode interface, the Al_2_O_3_ layer can suppress interfacial degradation by limiting oxygen scavenging from electrodes and reducing oxygen vacancy generation, as well as blocking charge injection across the interface, thereby improving endurance and cycling stability [139, 142]. When embedded within the HZO film, a thin Al_2_O_3_ layer can interrupt grain boundaries that would otherwise extend between the two electrodes, blocking trap‐assisted leakage paths and improving breakdown reliability and endurance [143, 144]. These results show that interlayers based on materials other than HfO_2_‐based compounds can also improve endurance in HZO capacitors, indicating that additional layer strategies serve as an important route for improving the cycling reliability of HfO_2_‐based ferroelectric devices.

While the above approach focuses on engineering the electrode‐ferroelectric interface, another additional layer strategy introduces a new interface within the capacitor stack. Liu et al. fabricated a 6 nm bilayer consisting of a 3 nm ZrO_2_ sublayer on a 3 nm HZO layer between W electrodes by ALD, followed by post‐deposition annealing at 450°C in N_2_ [145]. This structure retained about 88% of its maximum polarization up to 10^10^ cycles under 4 MV cm^−1^ without dielectric breakdown. The internal ZrO_2_/HZO interface suppresses charged defect migration and limits injected carrier transport, while the enhanced breakdown field strength of ultrathin layers contributes to the stable cycling. However, the switchable polarization decreased at a lower cycling field of 2.8 MV cm^−1^, since the non‐ferroelectric interfacial layer in such ultrathin films requires a higher operating field for full switching. While additional layer insertion can mitigate interfacial issues and enhance endurance, the resulting reliability also depends on how the ferroelectric film itself is fabricated, including the deposition sequence and initial defect distribution.

The deposition method directly influences the initial defect distribution, oxygen vacancy concentration, and interfacial redox state, all of which determine cycling endurance [65, 105, 146, 147]. Trinh et al. demonstrated that atomic layered modulation (ALM), in which two precursors are sequentially introduced within a single deposition cycle and subsequently reacted together using a common oxidant pulse, can enhance both ferroelectric performance and endurance compared to conventional ALD [136]. As schematically illustrated in Figure 7f, the ALM process relies on the difference in molecular size between tetrakis [dimethylamino]hafnium (TDMAH) and tetrakis[ethylmethylamino]zirconium (TEMAZ) precursors, enabling Hf and Zr atoms to be incorporated together within the same monolayer rather than forming separated sublayers. In addition, introducing a purge between the two precursor pulses removes weakly bound species and reaction residues from the surface before introduction of the second precursor. This process increases the fraction of the deposited film formed through stable chemisorbed species. As a result, the deposited films exhibit a denser microstructure, reduced oxygen vacancy concentration, and more homogeneous atomic‐scale mixing in which Hf and Zr atoms are positioned adjacent to each other within the same atomic plane. This closer spatial arrangement of these two cations in the as‐deposited state lowers the energetic barrier for the formation of the ferroelectric orthorhombic phase during post‐deposition annealing. As shown in Figure 7g, the 4.5 nm‐thick HZO film grown by ALM showed significantly higher *P_r_* than the ALD‐grown film and this enhancement was further pronounced compared to the thicker 9 nm films, owing to the improved ferroelectric orthorhombic phase fraction as a result of superior atomic‐scale mixing and crystallinity. The denser microstructure and reduced oxygen vacancy concentration in the ALM films are expected to suppress leakage current paths, leading to the lower leakage current during cycling. These results demonstrate that tuning the precursor sequence and the resulting defect during deposition can simultaneously improve both ferroelectric performance and cycling endurance in ultrathin HZO films.

A more fundamental approach involves scaling the ferroelectric layer thickness. Toprasertpong et al. reported that reducing thickness of the HZO from 9.5 to 4 nm increases number of cycle‐to‐breakdown by more than four orders of magnitude [148]. This improvement arises from the voltage‐dominated nature of dielectric breakdown, as opposed to electric field driven polarization switching. Under the same electric field, thinner films require a lower applied voltage. Consequently, electrons injected from the electrode carry less energy and inflict less damage on the ferroelectric layer, which delays the formation of conduction paths. Furthermore, the breakdown voltage remains nearly constant while the *V_c_* increases with thickness, reducing the film thickness increases the margin between *E_c_* and the breakdown field without altering the operating field scale. In addition, operating well below twice the *E_c_* promotes fatigue through charge redistribution, whereas operating well above this level accelerates dielectric breakdown through the formation of leakage current paths. Therefore, an operating field near twice the *E_c_* provides a practical balance between these two degradation modes. In practice, operating a 4 nm HZO at approximately twice the *E_c_* (1.2 MV cm^−1^) mitigates both fatigue and premature breakdown, yielding endurance on the order of 10^12^ cycles.

Across these strategies, the endurance of HfO_2_‐based ferroelectric capacitors can be strongly influenced by interfacial defect chemistry, microstructure, and the magnitude of the operating field relative to the *E_c_*. Interlayer engineering and deposition process optimization can enhance endurance by suppressing the generation and accumulation of defects associated with leakage path formation while simultaneously preserving the stability of the ferroelectric phase. Combining these approaches within a single device may further enhance the cycling reliability of HfO_2_‐based ferroelectrics toward the endurance levels required for embedded non‐volatile memory applications.

### 4.3 Interface and Multilayer Engineering for Fatigue‐Free Operation

**FIGURE 8:**
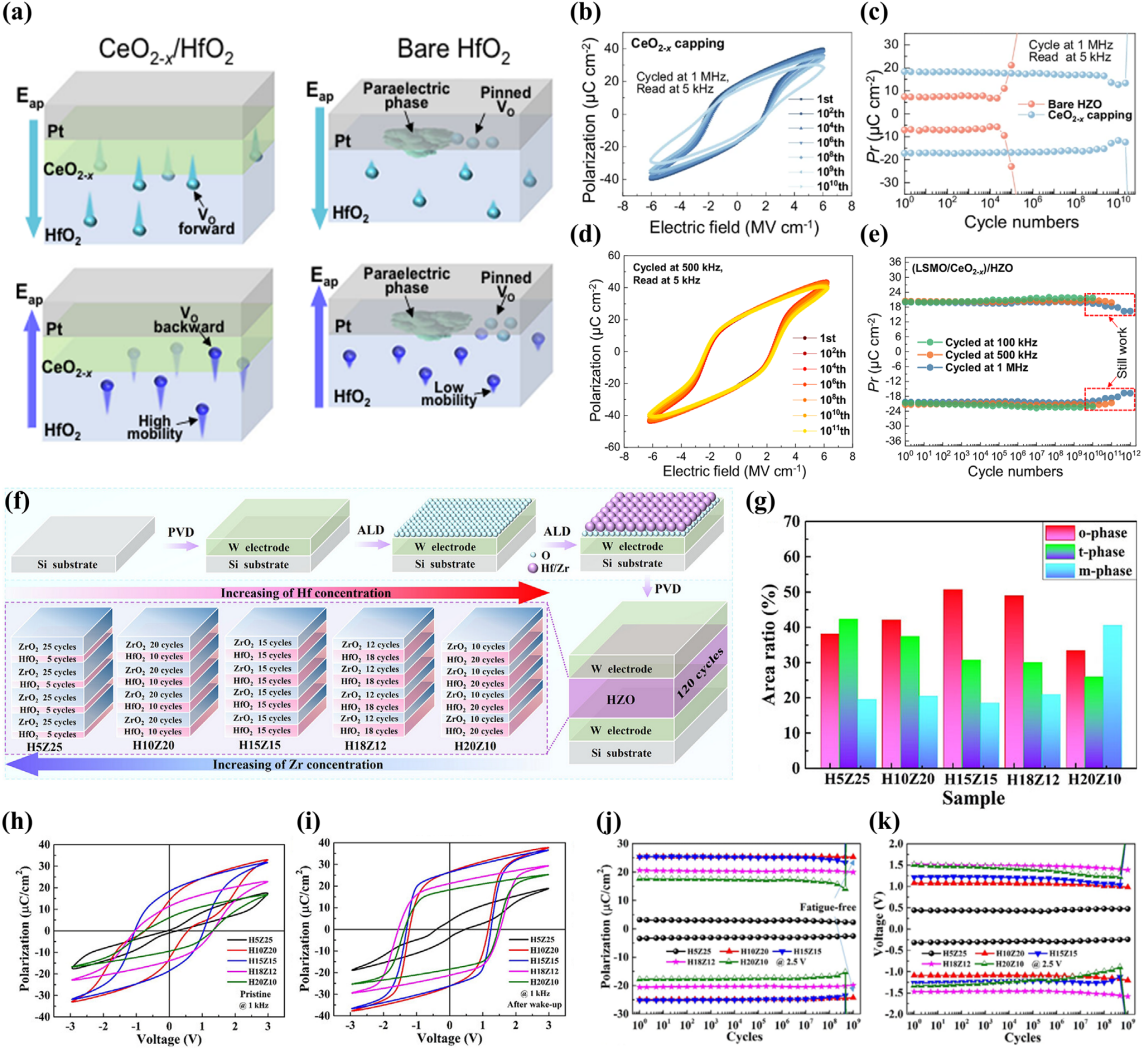
Fatigue‐free and high‐endurance operation in HZO capacitors through interface and multilayer engineering. (a) Schematic illustration of oxygen vacancy migration in CeO_2‐_ *_x_*/HfO_2_ and bare HfO_2_ capacitors, under applied electric fields. (b) P‐E hysteresis loops of the Pt/CeO_2‐_ *_x_*/HZO/LSMO capacitor with electrical cycling. (c) *P_r_* as a function of switching cycles for bare HZO and CeO_2‐_ *_x_*‐capped HZO capacitors. (d) P‐E hysteresis loops of the Pt/LSMO/CeO_2‐_ *_x_*/HZO/LSMO capacitor with fatigue‐free behavior up to 10^11^ cycles. (e) *P_r_* as a function of switching cycles for the Pt/LSMO/CeO_2‐_ *_x_*/HZO/LSMO capacitor cycled at different frequencies. Reproduced with permission [149]. Copyright 2025, Springer Nature Limited. (f) Schematic illustration of HZO multilayer capacitors with varying ZrO_2_ sublayer thickness and HfO_2_/ZrO_2_ cycle ratios. (g) Phase ratios of the orthorhombic (o‐), tetragonal (t‐) and monoclinic (m‐) phases extracted from GIXRD patterns for each multilayer composition. (h,i) P‐V hysteresis loops of HfO_2_‐ZrO_2_ multilayer capacitors in the pristine state and after wake‐up, respectively. (j,k) Evolution of *P_r_* and *V_c_* as a function of switching cycles for MFM capacitors with different HfO_2_‐ZrO_2_ stacking sequence, demonstrating near fatigue‐free endurance up to 10^9^ cycles. Reproduced with permission [150]. Copyright 2025 Wiley‐VCH GmbH.

From a reliability‐engineering perspective, suppressing imprint and dielectric loss benefits from symmetric interface design, balanced oxygen reservoirs, and minimized asymmetric charge trapping. These approaches reduce the internal bias field and non‐switching energy dissipation, thereby delaying fatigue onset and leakage‐driven breakdown. Beyond enhancing endurance during electrical cycling, recent studies have combined multiple interface and structural design strategies to achieve both high endurance and fatigue‐free operation while maintaining stable ferroelectric switching behavior [115, 151]. Zhou et al. proposed a multi‐dimensional interface engineering strategy that combines an oxygen‐active capping layer with a symmetric capacitor architecture to realize fatigue‐free cycling in pulsed laser deposition (PLD)‐grown HZO [149]. As schematically illustrated in Figure 8a, a coherent CeO_2‐_ *_x_* capping layer acts as an oxygen sponge that can reversibly accommodate oxygen vacancies through interfacial Ce^3+^/Ce^4+^ interconversion under alternating electric fields, thereby reducing defect accumulation at the electrode‐ferroelectric interface. First‐principles calculations showed that the CeO_2‐_ *_x_*/HfO_2_ heterointerface substantially lowers the oxygen vacancy diffusion barrier. In addition, the higher symmetry of the cubic CeO_2‐_ *_x_* also reduces both the polarization‐switching barrier and *E_c_*, consistent with the enhanced hysteresis loop of the Pt/CeO_2‐_ *_x_*/HZO/La_0.67_Sr_0.33_MnO_3_ (LSMO) capacitor shown in Figure 8b. In contrast, bare HZO lacks an oxygen reservoir layer, leaving the Pt/HZO interface as a terminal site for oxygen accumulation during cycling. The resulting change in the local oxygen chemical potential at the ferroelectric/electrode interface stabilizes the energetically favorable non‐polar monoclinic phase, promoting interfacial phase transition and degradation of ferroelectric switching. However, as shown in Figure 8c, the CeO_2‐_ *_x_* layer alone could not fully achieve fatigue‐free behavior, with polarization degradation observed after approximately 10^9^ cycles. This limitation is attributed to net directional defect migration driven by the built‐in imprint field, which gradually depletes the oxygen sponge capacity of the CeO_2‐_ *_x_* layer. In an asymmetric capacitor structure, the built‐in imprint field superimposes on the externally applied bipolar field, producing asymmetric effective electric fields during opposite half‐cycles. Consequently, oxygen vacancies migrate at different velocities and migration distances during forward and reverse switching processes. This asymmetric motion leads to a net directional drift during repeated cycling, as evidenced by the progressive decrease in the Ce^3+^ fraction after extended cycling. Furthermore, comparison among representative HfO_2_‐based ferroelectric capacitors revealed that stronger imprint fields are generally associated with earlier fatigue onset, supporting the interpretation that the built‐in field drives directional defect migration. To suppress this directional drift, an additional LSMO layer was introduced on top of the CeO_2‐_ *_x_* to form a symmetric Pt/LSMO/CeO_2‐_ *_x_*/HZO/LSMO structure. The top LSMO restores structural symmetry and suppresses the directional defect drift caused by the imprint field, while simultaneously acting as a secondary oxygen reservoir together with the CeO_2‐_ *_x_* layer, thereby forming a bilayer oxygen sponge configuration. As shown in Figure 8d,e, this combined design enables stable fatigue‐free switching with less than 5% polarization loss over 10^11^ cycles, maintaining endurance beyond 10^12^ cycles, demonstrating that fatigue‐free operation requires simultaneous control of interfacial defect dynamics and macroscopic field asymmetry.

Multilayer structural engineering has also emerged as an effective strategy for achieving near‐fatigue‐free operation in HfO_2_‐based ferroelectrics [152, 153, 154]. Yan et al. demonstrated that zirconium‐rich HfO_2_‐ZrO_2_ multilayer films fabricated by alternating ALD subcycles can achieve both high polarization and reduced *E_c_* through deliberate control of the sublayer stacking sequence [150]. The fabricated samples were labelled as H*_x_* Z*_y_* according to the number of HfO_2_(*x*) and ZrO_2_(*y*) ALD subcycles in each stacking unit. The total number of deposition cycles was maintained at 120 for all configurations. As illustrated in Figure 8f, systematic variation of the HfO_2_/ZrO_2_ cycle ratio enables precise tuning of the local Hf and Zr content within the multilayer stack. Increasing the Zr fraction shifts the phase stability away from the ferroelectric orthorhombic phase, which is typically favored near the equimolar composition, toward the tetragonal antiferroelectric‐like phase. At the same time, Zr‐rich sublayers introduce tensile strain into adjacent HfO_2_ layers, lowering the nucleation barrier for ferroelectric phase formation. Grazing incidence X‐ray diffraction (GIXRD) analysis (Figure 8g) reveals that increasing ZrO_2_ sublayer thickness progressively increases the tetragonal phase fraction while reducing the monoclinic phase, with the H15Z15 composition exhibiting the highest orthorhombic phase fraction. Aberration‐corrected STEM further confirms that local interlaminar diffusion at the sublayer interfaces forms a compositionally graded (Hf, Zr)O_2_ region, which stabilizes a mixed orthorhombic and tetragonal phase configuration. As shown in Figure 8h,i, the optimized H10Z20 configuration exhibits a pinched, antiferroelectric‐like hysteresis loop in the pristine state that evolves into a well‐saturated ferroelectric loop after wake‐up, reflecting a field‐driven tetragonal to orthorhombic phase transition. Furthermore, DFT calculations indicate that an applied electric field induces a unit‐cell contraction, resulting in increased overlap between the tetragonal and orthorhombic free energy landscapes. After removal of the field, the system relaxes preferentially along the orthorhombic pathway, explaining the pronounced increase in *P_r_* observed after the wake‐up process. This compositionally graded interface reduces the switching field while maintaining high *P_r_*, which in turn translates into near fatigue‐free endurance with negligible polarization degradation and stable *V_c_* after 10^9^ cycles, as shown in Figure 8j,k. In addition, the relatively low annealing temperature used in this study likely suppresses excessive defect formation during crystallization, further contributing to the reduced fatigue rate. These results indicate that reducing the *E_c_* through interlaminar diffusion, combined with favorable phase stabilization in the multilayer architecture, provides an effective structural route toward highly fatigue‐resistant operation in HfO_2_‐based ferroelectrics.

Similarly, superlattice structures have recently been studied as a strategy toward fatigue‐free and high endurance HfO_2_‐based ferroelectrics, where alternating sublayers and their initial interfaces are exploited to stabilize the ferroelectric phase and suppress degradation by defects. Liang et al. demonstrated that combining an HfO_2_‐ZrO_2_ superlattice with ZrO_2_ as the initial layer and optimized annealing yields nearly fatigue‐free polarization behavior [151]. A 10 nm‐thick stack consisting of four periods of a 1.25 nm ZrO_2_ and 1.25 nm HfO_2_ sublayers was grown by thermal ALD between TiN electrodes, followed by rapid thermal annealing in N_2_ at 350–600°C. The 600°C post‐metal annealing (PMA) sample maintained polarization variation below 9% up to 10^11^ cycles, while lower temperature samples exhibited progressive degradation during cycling. This behavior likely reflects two complementary contributions. High annealing temperatures produce more uniform and well‐aligned grains, thereby lowering the density of structural inhomogeneities that mediate polarization redistribution under cycling. In addition, the alternating ZrO_2_‐HfO_2_ sublayers and internal interfaces may obstruct rapid vacancy migration and charge transport along grain boundaries and other defect paths, and also limit charge injection from the electrode into the ferroelectric region. These contributions may mitigate the gradual accumulation of charged defects near the interface, showing that careful annealing control combined with a layered film structure can improve fatigue resistance in HfO_2_‐based ferroelectric capacitors.

Overall, these studies reflect a broader shift toward fatigue‐free design strategies that reduce polarization loss during the entire cycling lifetime. The suppression of net oxygen vacancy drift and stabilization of the ferroelectric phase under repeated cycling are essential for achieving fatigue‐free behavior. Therefore, integrating interfacial defect engineering with structural engineering within scalable, CMOS‐compatible fabrication process is expected to be a promising pathway for achieving high endurance and fatigue‐free operation in HfO_2_‐based ferroelectrics.

### 4.4 Various Approaches for Achieving High Endurance

**FIGURE 9:**
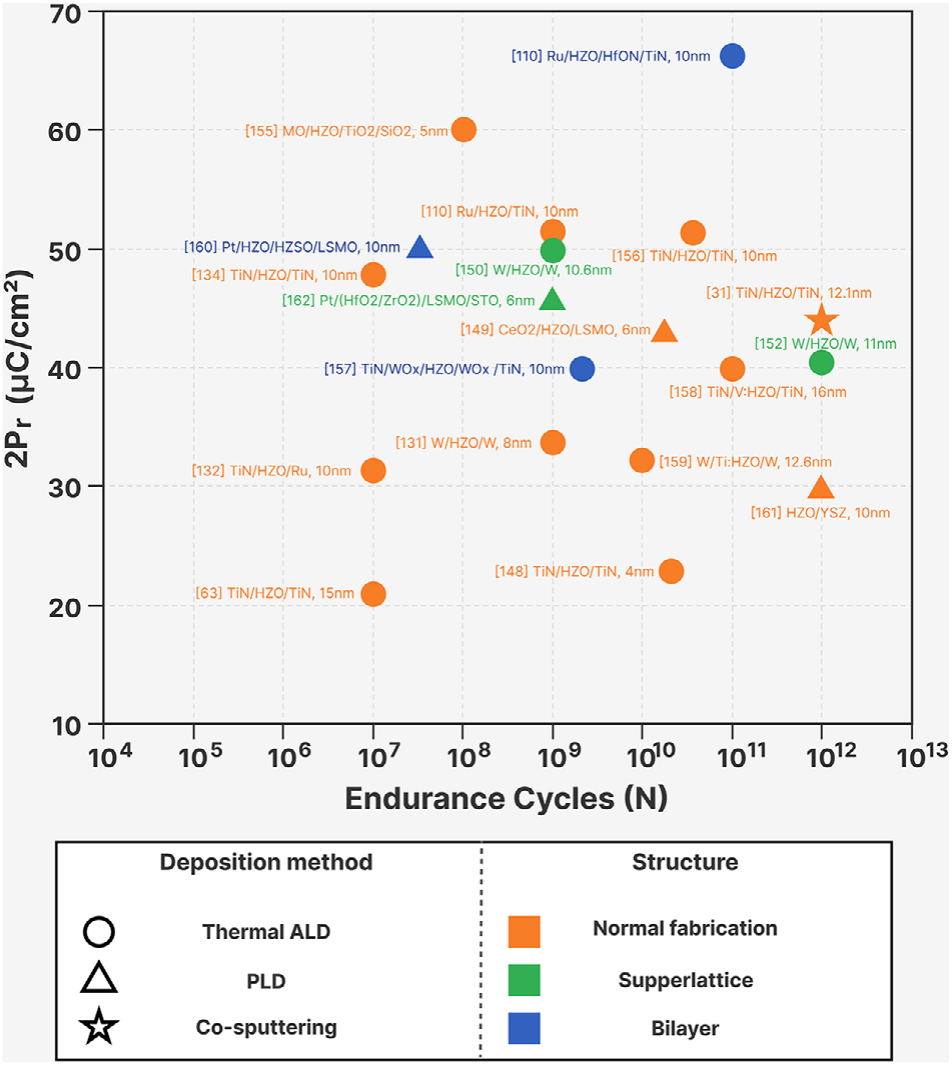
Comparison of 2*P_r_* versus endurance cycles for HfO_2_‐based ferroelectrics, shown in Table 2 with different deposition methods and device structures. Symbols and colors indicate fabrication methods (thermal ALD, PLD, co‐sputtering) and structures (normal, superlattice, bilayer) respectively.

Table 2 and Figure 9 provide a comprehensive overview of endurance performance reported for representative HfO_2_‐based ferroelectrics with various fabrication approaches. Because these endurance values were measured under different cycling frequencies, applied fields, and waveform conditions, they should not be compared solely by the nominal cycle number. Instead, the cycling protocol should be considered together with material composition, electrode configuration, and device structure. The data illustrates that endurance is strongly dependent on device structure rather than material composition alone, with reported values ranging from 10^7^ to 10^12^ cycles depending on electrode configuration, interlayer design, and structural engineering strategies. Notably, devices incorporating reactive electrodes, oxygen‐accommodating interlayers, or multilayer/superlattice structures consistently achieve endurance in the 10^9^–10^12^ cycle range, validating the effectiveness of the strategies discussed above. Even for the same nominal composition such as Hf_0.5_Zr_0.5_O_2_, endurance can differ depending on the fabrication processes and structure, which highlights that the process and the structural engineering are as important as the material composition for device reliability. This summary provides a practical reference for guiding future device design toward reliable long‐term operation.

**TABLE 2:** Overview of endurance performance in HfO_2_‐based ferroelectric capacitors categorized by deposition method and device structure. The comparison highlights the effectiveness of various engineering approaches, including electrode selection, interlayer insertion, and multilayer structures, in improving endurance.

Deposition method	Material	Thickness	Doping	Structure	Endurance	Frequency	Applied field	Waveform	References
Thermal ALD	Hf_0.5_Zr_0.5_O_2_	10 nm	Zr	Ru/HZO/HfON/TiN	10^11^	100 kHz	±4.0 MV cm^−1^	Not reported	[110]
Thermal ALD	Hf_0.5_Zr_0.5_O_2_	10 nm	Zr	Ru/HZO/TiN	10^9^	100 kHz	±4.0 MV cm^−1^	Not reported	[110]
Thermal ALD	Hf_0.5_Zr_0.5_O_2_	5 nm	Zr	Mo/HZO/TiO_2_/SiO_2_ (MFIS)	10^8^	50 kHz	+2.5 MV cm^−1^ / ‐3.0 MV cm^−1^	Bipolar rectangular	[155]
Thermal ALD	Hf_0.5_Zr_0.5_O_2_	10 nm	Zr	TiN/HZO/TiN	6 × 10^10^	100 kHz	±3.0 MV cm^−1^	Bipolar rectangular	[156]
Thermal ALD	Hf_0.5_Zr_0.5_O_2_	8 nm	Zr	W/HZO/W	10^9^	50 kHz	±1.5 MV cm^−1^	Bipolar rectangular	[131]
Thermal ALD	Hf_0.5_Zr_0.5_O_2_	10 nm	Zr	TiN/HZO/TiN	10^7^	100 kHz	±3.0 MV cm^−1^	Bipolar rectangular	[134]
Thermal ALD	Hf_0.5_Zr_0.5_O_2_	10 nm	Zr	TiN/WO*_x_*/HZO/WO*_x_*/TiN	3.2 × 10^9^	100 kHz	±2.0 MV cm^−1^	Bipolar rectangular	[157]
Thermal ALD	Hf_0.5_Zr_0.5_O_2_	10 nm	Zr	TiN/HZO/Ru	10^7^	50 kHz	±2.5 MV cm^−1^	Bipolar rectangular	[132]
Thermal ALD	Hf_0.5_Zr_0.5_O_2_	4 nm	Zr	TiN/HZO/TiN	3 × 10^10^	200 kHz	±3.0 MV cm^−1^	Bipolar trapezoid (rise, fall time 0.5 µs)	[148]
Thermal ALD	Hf_0.5_Zr_0.5_O_2_	15 nm	Zr	TiN/HZO/TiN	10^7^	500 kHz	±1.8 MV cm^−1^	Not reported	[63]
Thermal ALD	Hf_0.5_Zr_0.5_O_2_	16 nm	Zr, V	TiN/V:HZO/TiN	10^11^	1 MHz	±3.0 MV cm^−1^ ±3.25 MV cm^−1^	Not reported	[158]
Thermal ALD	Hf_0.5_Zr_0.5_O_2_	12.6 nm	Zr, Ti	TiN/Ti:HZO/TiN/W	10^10^	500 kHz	±1.59 MV cm^−1^	Not reported	[159]
Thermal ALD	Superlattice (HZO‐ZrO_2_)	11 nm	Zr	W/HZO/W	10^12^	5 MHz	±2.72 MV cm^−1^	Not reported	[152]
Thermal ALD	Superlattice (H10Z20)	10.6 nm	Zr	W/HZO/W	10^9^	1 MHz	±2.35 MV cm^−1^	Bipolar rectangular	[150]
PLD	Hf_0.5_Zr_0.45_Sm_0.05_O_1.61_	10 nm	Zr, Sm	Pt/HZO(8 nm)/HZSO(2 nm)/LSMO	5 × 10^7^	500 kHz	±5.0 MV cm^−1^	Bipolar rectangular	[160]
PLD	Hf_0.5_Zr_0.5_O_2_	6 nm	Zr	CeO_2_/HZO/LSMO	2 × 10^10^	1 MHz	±6.0 MV cm^−1^	Bipolar rectangular	[149]
PLD	Hf_0.5_Zr_0.5_O_2_	10 nm	Zr	HZO/YSZ	10^12^	10 MHz	±10.0 MV cm^−1^	Not reported	[161]
PLD	Superlattice HfO_2_/ZrO_2_ (*n* = 3)	6 nm	Zr	Pt/(HfO_2_/ZrO_2_)/LSMO/STO	10^9^	1 MHz	±6.67 MV cm^−1^	Bipolar rectangular	[162]
Co‐sputtering	HfZr_(1+_ *_x_*_)_O_2_	12.1 nm	Zr	TiN/HZO/TiN	10^12^	1 MHz	±1.25 MV cm^−1^	Bipolar rectangular	[31]

Beyond the protocol information summarized in Table 2 and Figure 9, future endurance comparisons should also include finite‐pulse descriptors such as *Q_sw_(t_p_)*, characteristic switching time, switching‐current evolution, switching‐field distribution, and switching probability under fixed pulses. These descriptors are essential for distinguishing devices that merely retain large quasi‐static *P_r_* from those that maintain deterministic, low‐energy, and high‐speed switching during cycling.

## 5 Impact of Reliability on Device Metrics and Emerging Applications

**FIGURE 10:**
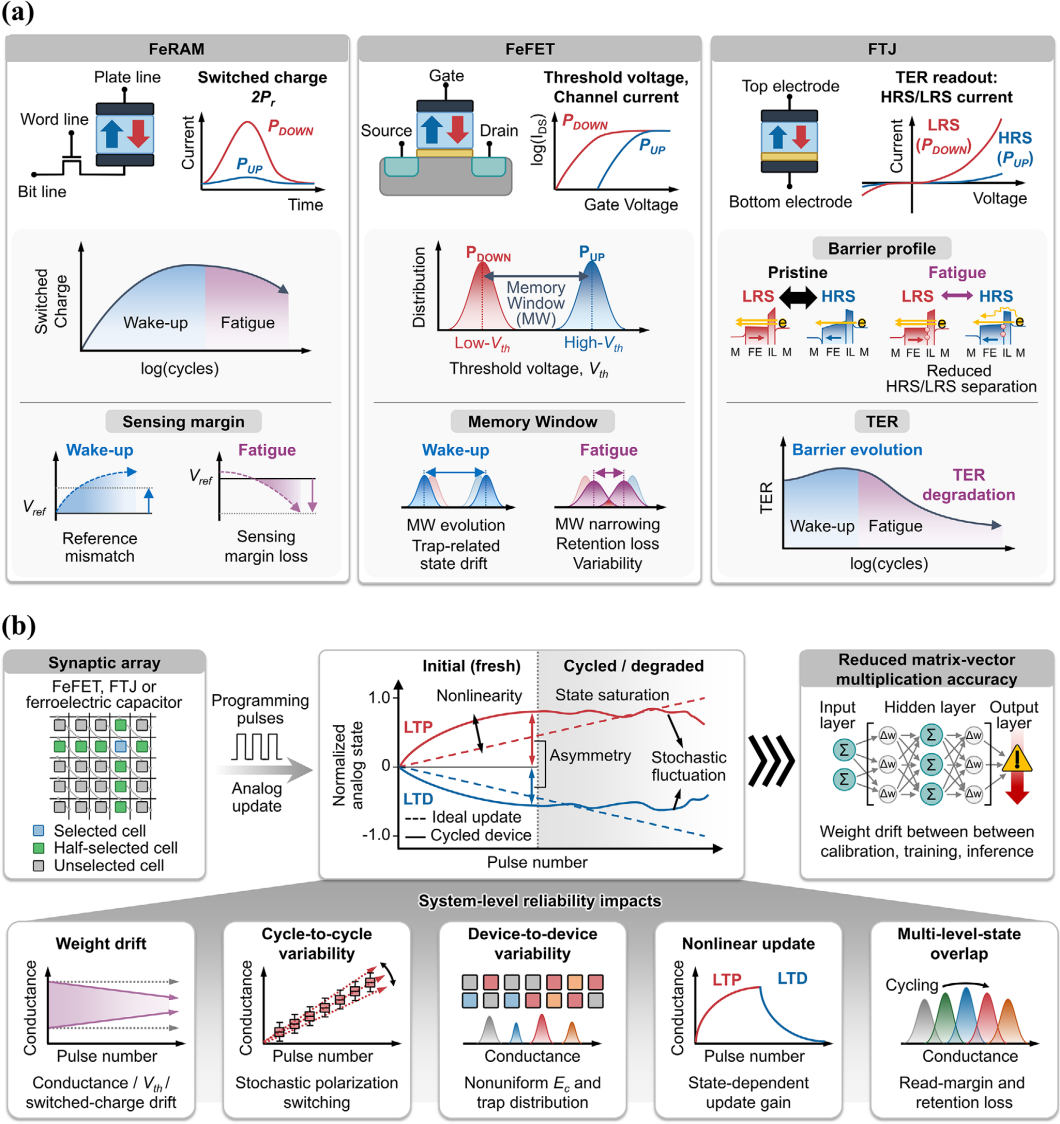
Device‐ and system‐level impact of defect‐controlled switching dynamics during wake‐up and fatigue in HfO_2_‐based ferroelectrics. (a) Representative FeRAM, FeFET, and FTJ architectures and their device‐level reliability metrics, including *Q_sw_*/sensing margin, *V_th_*/MW, and TER/HRS/LRS readout. (b) Impact on LIM/CIM/neuromorphic systems, where cycling‐induced pulse‐to‐state distortion leads to weight drift, variability, nonlinear update, multi‐level‐state overlap, and reduced matrix‐vector multiplication accuracy.

The wake‐up and fatigue mechanisms discussed above are commonly identified using MFM capacitor structures, where polarization hysteresis, *P_r_*, leakage current, and endurance cycles provide direct indicators of material‐level reliability. However, in practical HfO_2_‐based devices, the same microscopic processes are translated into different performance metrics depending on the device architecture and readout scheme. As summarized in Figure 10a, wake‐up and fatigue appear as changes in switched charge (*Q_sw_*) and sensing margin in FeRAM, threshold‐voltage distribution and memory window (MW) in FeFETs, and high‐resistance states (HRS)/low‐resistance states (LRS) current separation and tunneling electroresistance (TER) in FTJs. Their influence becomes even more complex in logic‐in‐memory (LIM), computing‐in‐memory (CIM), and neuromorphic systems, where repeated programming must preserve stable analog pulse‐to‐state characteristics. As illustrated in Figure 10b, cycling‐induced drift, variability, nonlinear updates, and multi‐level‐state overlap can degrade analog‐state retention and reduce matrix‐vector multiplication accuracy. These device‐level consequences are governed not only by quasi‐static *P_r_*, but also by finite‐pulse switching dynamics. Defect redistribution, trapped charge, domain‐wall pinning, phase evolution, and leakage‐path formation reshape local switching‐field and switching‐time distributions, thereby changing *Q_sw_(t_p_)*, switching probability, threshold voltage (*V_th_*)/MW distribution, resistance‐state formation, and analog update response under fixed programming pulses. Therefore, evaluating the practical reliability of HfO_2_‐based ferroelectrics requires connecting capacitor‐level cycling physics with the specific electrical metrics that determine each memory and computing architecture [65, 163, 164].

### 5.1 Device‐Level Perspectives: From Capacitors to Transistors

While MFM capacitors provide fundamental insights into polarization switching and defect dynamics, practical reliability must be assessed in the context of specific device architectures, including capacitor‐based FeRAM, FeFETs, and FTJs. As illustrated in Figure 10a, the same microscopic processes, such as oxygen‐vacancy redistribution, charge injection and trapping, domain‐wall pinning, phase evolution, and leakage‐path formation, are projected onto different performance metrics depending on the readout scheme and device geometry.

In capacitor‐based FeRAM, typically implemented as a 1T1C cell using an MFM ferroelectric capacitor, the primary readout variable is the *Q_sw_*. Under practical pulses, the relevant metric is *Q_sw_* (*t_p_*), rather than only quasi‐static *P_r_*, because fatigue‐induced broadening of switching times can reduce sensed charge even before a large *P_r_* loss is observed. Wake‐up can initially increase the switchable polarization and enlarge the sensing margin. However, if wake‐up proceeds during circuit operation after array calibration, the time‐dependent evolution of *Q_sw_* can induce reference‐level drift, cell‐to‐cell variability, and read‐margin instability. Fatigue has a particularly detrimental impact on device operation because the reduction in *P_r_* or *Q_sw_* shrinks the sensing margin, required for reliable read operation. In addition, cycling‐induced imprint, leakage‐current increase, and incomplete polarization reversal can degrade retention and disturb immunity. These issues are particularly important because conventional FeRAM readout is destructive and requires a write‐back operation after reading [15, 165, 166, 167].

FeFETs, typically based on metal‐ferroelectric‐insulator‐semiconductor (MFIS) or metal‐ferroelectric‐metal‐insulator‐semiconductor (MFMIS) gate stacks, present reliability challenges that are distinct from simple MFM capacitors [168]. In FeFETs, the memory state is read through the *V_th_* or channel current, and the MW is determined not only by ferroelectric polarization but also by electrostatic coupling through the interfacial dielectric and semiconductor channel. In FeFETs, finite‐pulse switching kinetics are convoluted with charge trapping and semiconductor electrostatics; broadened local switching‐time distributions can appear as wider *V_th_* distributions after identical program/erase pulses, while trap‐assisted internal‐bias evolution can produce cycle‐dependent program/erase asymmetry. Therefore, wake‐up does not simply appear as an increase in *P_r_*. It can also modify the *V_th_* distribution through internal‐bias relaxation, depolarization‐field changes, and trap charging. During repeated program/erase cycling, charge injection and trap generation at the ferroelectric/interlayer/semiconductor interfaces can screen the ferroelectric polarization and induce asymmetric *V_th_* shifts. Consequently, fatigue in FeFETs is manifested as MW narrowing, *V_th_* drift, degraded retention, increased device‐to‐device variability, and, in severe cases, gate‐stack degradation or leakage increase. Importantly, a FeFET may exhibit substantial MW degradation even when the corresponding MFM capacitor shows relatively stable polarization, because interfacial charge trapping and semiconductor electrostatics provide additional degradation pathways. These effects are particularly relevant for oxide‐semiconductor‐channel FeTFTs, where polarization compensation, interfacial defect control, oxygen‐vacancy suppression, and gate‐stack electrostatics strongly affect MW, endurance, and retention [169, 170, 171].

In FTJs, ultrathin HZO or HfO_2_‐based layers serve as the ferroelectric tunneling barrier or as part of an asymmetric tunneling barrier stack. The device state is read through the tunneling current, and the TER ratio depends sensitively on the polarization‐dependent barrier height, barrier width, interfacial screening, and defect distribution. In FTJs, finite‐pulse switching kinetics determine the spatial uniformity and temporal stability of the polarization‐dependent tunneling barrier; partial or nonuniform switching can enable multi‐level states, but defect‐assisted tunneling and oxygen‐vacancy redistribution can broaden or drift these states during cycling. Wake‐up can therefore change the resistance states by modifying both the polarization state and the internal barrier profile. Under prolonged cycling, oxygen‐vacancy redistribution, trap generation, and interfacial charge accumulation can enhance trap‐assisted tunneling and distort the potential barrier. These changes reduce the TER, shift the HRS and LRS, and narrow the read margin. For multi‐level FTJ operation, fatigue‐induced resistance drift and distribution broadening can cause overlap between adjacent states, thereby degrading retention and limiting high‐density storage [172, 173, 174, 175, 176].

These device‐level differences indicate that capacitor‐level endurance alone is insufficient to predict practical reliability. FeRAM requires stable *Q_sw_* and sensing margin, FeFETs require stable MW and *V_th_* distributions, and FTJs require stable tunneling‐barrier profiles and TER ratios. Table 3 summarizes how wake‐up and fatigue are translated into device‐specific performance metrics in FeRAM, FeFET, FTJ, and analog in‐memory computing architectures.

**TABLE 3:** Device‐level impact of wake‐up and fatigue in HfO_2_‐based ferroelectric devices and emerging computing architectures.

Device/application	Representative structure	Main readout variable	Wake‐up impact	Fatigue impact	Unique reliability issue
FeRAM	1T1C cell with MFM ferroelectric capacitor	*Q_sw_*, *2P_r_*	Increase of switchable polarization, possible reference‐level drift if wake‐up occurs after calibration	Reduction of *Q_sw_* and sensing margin, imprint, leakage increase, retention degradation	Destructive readout, write‐back operation, sense‐amplifier margin
FeFET	MFIS or MFMIS gate stack	*V_th_*, MW, channel current	MW evolution, *V_th_* distribution shift due to internal‐bias relaxation and trap charging	MW narrowing, *V_th_* drift, retention loss, variability, gate‐stack degradation	Interface charge trapping, interfacial dielectric degradation, semiconductor electrostatics
FTJ	Ultrathin HZO‐based tunneling barrier	TER ratio, high‐/low‐resistance states, read current	Barrier‐profile evolution, resistance‐state drift	TER degradation, ON/OFF resistance overlap, trap‐assisted tunneling, retention loss	Barrier integrity, oxygen‐vacancy redistribution, multi‐level‐state separation
LIM, CIM, neuromorphic systems	FeFET, FTJ, or capacitor arrays	Analog conductance, *V_th_*, *Q_sw_*	Time‐dependent pulse‐to‐state transfer function, degraded update linearity	Weight drift, stochastic updates, increased variability, multi‐level‐state overlap	LTP/LTD symmetry, analog precision, inference accuracy, calibration stability

While Table 3 summarizes device‐specific reliability consequences, a complementary set of finite‐pulse descriptors is needed to connect defect/phase evolution with practical switching operation. Table 4 summarizes representative switching‐kinetics descriptors that can be monitored together with conventional reliability metrics such as *P_r_*, *E_c_*, leakage current, and endurance cycles.

**TABLE 4:** Representative finite‐pulse switching‐kinetics descriptors for correlating wake‐up/fatigue mechanisms with practical device reliability.

Kinetic descriptor	Cycling‐induced signature	Practical relevance
Pulse‐width‐dependent switched charge, *Q_sw_* (*t_p_*)	Wake‐up increases *Q_sw_* (*t_p_*) under short pulses; fatigue causes incomplete switching.	FeRAM sensing margin, FeFET MW formation, write energy
Characteristic switching time, *τ_50_/τ_90_*	Wake‐up can shorten or narrow the switching‐time distribution; fatigue delays or broadens it	Write latency, minimum pulse width, timing margin
Switching‐current peak position and width	Wake‐up sharpens or symmetrizes peaks; fatigue shifts, broadens, or splits peaks.	Early kinetic diagnosis, pulse optimization
Switching‐field or *E_c_* distribution	Internal‐bias relaxation narrows/shifts the distribution; imprint/trapping broadens or shifts it.	*V_th_* distribution, cell variability, switching asymmetry
Switching probability under fixed pulses	Wake‐up improves deterministic switching; fatigue increases stochastic or failed switching.	Write‐error rate, verify burden, array yield
Pulse‐to‐state transfer function	Identical pulses produce drifting or nonlinear state updates during cycling.	CIM/neuromorphic update precision, multi‐level‐state separation
NLS switching‐time distribution width, w or σlogτ	Wake‐up can reduce w by homogenizing internal bias and depinning domains; residual or generated defects, grain boundaries, and phase coexistence increase w by broadening local nucleation barriers.	Write‐error tail, device‐to‐device variability, verify burden, stochastic switching

### 5.2 Circuit‐Level Kinetic Engineering: Wake‐Up Pre‐Conditioning, Pulse Scaling, and High‐Field Operation

The kinetic interpretation above also clarifies why electrical pre‐conditioning should be evaluated not simply by the number of wake‐up cycles, but by how the switching‐field and switching‐time distributions are shifted into the circuit operating window. Recent dynamic random‐access memory (DRAM)‐oriented studies further illustrate how electrical pre‐conditioning protocols can be evaluated in a circuit‐relevant manner. Shin et al. reported a Y‐doped Hf_0.5_Zr_0.5_O_2_ field‐induced‐ferroelectric DRAM capacitor in which simple prolonged high‐field cycling was not ideal because cycling at 6 MV cm^−1^ for 10^7^ cycles lowered the switching field but also induced significant remanent‐polarization increase and leakage‐current degradation. To mitigate this trade‐off, they introduced a stepwise cycling protocol consisting of 6 MV cm^−1^ for 10^5^ cycles, 5 MV cm^−1^ for 10^5^ cycles, and 4 MV cm^−1^ for 10^7^ cycles. This amplitude‐stepped protocol shifted the field‐induced switching distribution into the 0.8 V operating range while suppressing excessive *P_r_*, hysteresis loss, and J degradation, yielding *k* ∼ 68 and equivalent oxide thickness (EOT) ∼ 0.31 nm in a 5.5 nm Y:Hf_0.5_Zr_0.5_O_2_ film that satisfied the DRAM leakage criterion [177].

The same study also translated the laboratory pulse conditions into a DRAM‐relevant time scale. By normalizing the measurement pulse duration using a resistance‐capacitance (RC) ratio of approximately 1351 between the experimental setup and a practical DRAM capacitor, a 20 µs measurement pulse was estimated to correspond to a DRAM time scale of approximately 14.8 ns, within the 10–20 ns DRAM read/write window. The 100 kHz triangular cycling pulse used for stepwise cycling was similarly estimated to correspond to approximately 7.4 ns on the DRAM time scale. Based on a 16 Gb DRAM architecture, the total time required for preconditioning all cells was estimated to be approximately 410 s, whereas reconditioning with 10^6^ cycles would require approximately 41 s. These results suggest that limited electrical pre‐conditioning or reconditioning may be practical when embedded into wafer‐level testing, package‐level burn‐in, initial product programming, or idle‐bank operation [177]. However, this example should be interpreted as a carefully engineered DRAM‐specific protocol rather than as evidence that prolonged wake‐up cycling is generally compatible with all HfO_2_‐based devices. In FeFETs and other gate‐stack devices, additional constraints from interfacial dielectrics, semiconductor electrostatics, charge trapping, and gate leakage must be considered [170, 171, 178].

### 5.3 Implications for Emerging Paradigms: LIM, CIM, and Neuromorphic Computing

Beyond conventional digital storage, HfO_2_‐based FeFETs, FTJs, and capacitor arrays are being explored as nonvolatile elements for LIM, CIM, and neuromorphic computing. In these systems, the device state is not limited to binary information. Conductance, threshold voltage, or *Q_sw_* must often be updated in small and reproducible increments to represent analog synaptic weights or multi‐level memory states. As illustrated in Figure 10b and summarized in Table 3, the reliability requirement is therefore more stringent than simple binary endurance because the pulse‐to‐state transfer function must remain stable over many update cycles [173, 179, 180, 181].

Wake‐up is particularly problematic for analog operation because it makes the response to identical programming pulses time‐dependent. During the wake‐up regime, oxygen vacancy redistribution and internal‐bias relaxation continuously modify the effective *E_c_* distribution. As a result, programming pulses optimized for the pristine state may produce progressively different changes in conductance, *V_th_*, or *Q_sw_*. This directly degrades the linearity and symmetry of long‐term potentiation and depression, increases device‐to‐device variation, and reduces the accuracy of open‐loop weight updates [46, 68, 96, 179].

Fatigue introduces an additional source of instability. Repeated cycling generates or redistributes defects, enhances charge trapping, and pins domain walls or phase boundaries. For analog synaptic devices, this can transform gradual polarization modulation into stochastic switching events, producing abrupt conductance jumps, increased cycle‐to‐cycle variability, and stochastic polarization jumps. In CIM arrays, such drift and variability reduce the accuracy of matrix‐vector multiplication because the programmed weight values can change between calibration, training, and inference. For multi‐level operation, fatigue‐induced depolarization fields, charge trapping, and leakage‐current increase can broaden the distributions of intermediate states and cause overlap between adjacent levels, thereby reducing read margin and retention [46, 91, 96, 182].

These issues indicate that the system‐level metrics listed in Table 3, including analog‐state retention, update variability, and pulse‐to‐state stability, are as important as conventional binary endurance for reliable in‐memory computing. Wake‐up‐free or fast‐wake‐up HfO_2_‐based stacks, fatigue‐resistant interfaces, and low‐*E_c_* designs are therefore essential device‐level strategies. At the circuit and system levels, write‐verify schemes, adaptive pulse programming, calibration/reference tracking, and training‐aware compensation may further mitigate residual wake‐up and fatigue effects [183, 184, 185, 186]. Therefore, pulse‐to‐state stability should be evaluated together with switching‐time‐distribution evolution, because wake‐up and fatigue can change the update step produced by identical programming pulses.

## 6 Summary and Outlook

In this review, we discussed the physical origins and mechanisms of wake‐up and fatigue in HfO_2_‐based ferroelectrics, along with experimentally demonstrated strategies for improving device endurance. Wake‐up behavior is generally attributed to the redistribution of pre‐existing oxygen vacancies and associated field‐induced phase transitions, whereas fatigue is primarily driven by the generation of additional defects and charge trapping, which can lead to domain pinning under prolonged cycling. Based on these mechanistic insights, various methodologies for enhancing reliability have been demonstrated, including composition and doping control, process optimization, electrode and interlayer design, and multilayer structural engineering. These strategies enable controlled wake‐up behavior and extended endurance in optimized device structures.

Despite these advances, several fundamental challenges remain unresolved. One of these challenges is understanding the long‐term cycling reliability of the polar rhombohedral phase. It has attracted increasing attention as a promising pathway for wake‐up‐free operation, because it exhibits weak or negligible wake‐up behavior. However, most studies to date, have primarily focused on its ferroelectric and wake‐up properties, whereas its fatigue behavior under prolonged cycling has received less attention than that of the orthorhombic phase. In particular, the fatigue mechanism of the rhombohedral phase and the associated phase evolution during electrical cycling remain poorly understood. Furthermore, it is unclear whether phase transitions between the rhombohedral and orthorhombic phases occur during repeated cycling and, if so, how such transformations influence fatigue and endurance behavior. Addressing these questions will be necessary for realizing wake‐up‐free, high endurance, and reliable HfO_2_‐based ferroelectric devices.

Another challenge concerns the reliability of endurance predictions, which remains limited as both wake‐up saturation and fatigue progression are sensitive to cycling frequency and field amplitude. Therefore, a comprehensive understanding of device endurance requires addressing these phenomena. A design strategy that integrates precise defect control with interface and structural engineering is expected to play an important role in achieving both wake‐up‐free operation and fatigue‐free endurance in next‐generation devices.

**FIGURE 11:**
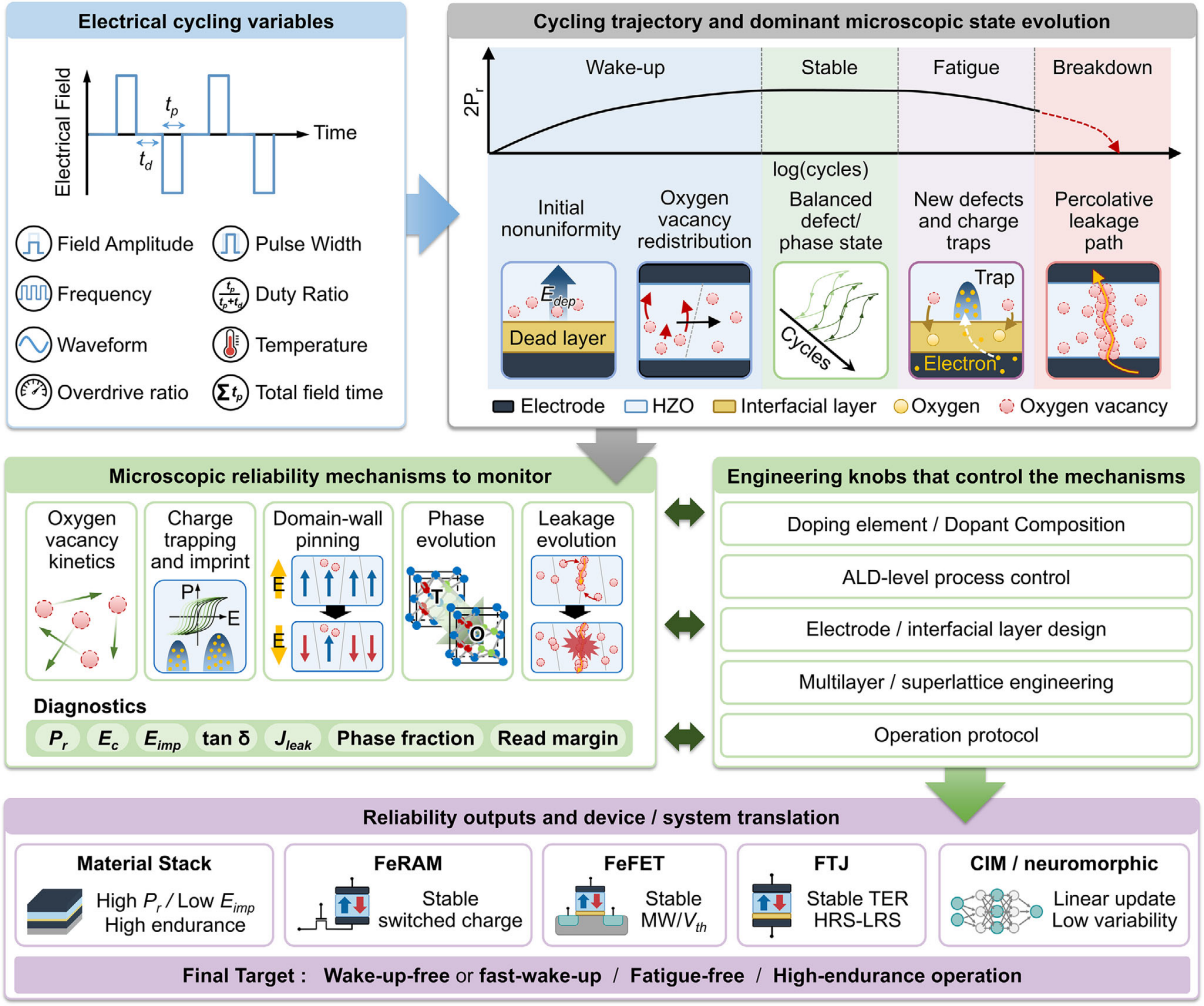
Integrated reliability‐design roadmap for reliable electrical cycling in HfO_2_‐based ferroelectrics. The roadmap links electrical cycling variables, microscopic state evolution, reliability mechanisms, engineering knobs, and device/system targets, providing design guidelines for wake‐up‐free, fatigue‐resistant, and high‐endurance operation.

As summarized in the integrated reliability‐design roadmap in Figure 11, future reliability‐improvement strategies are expected to move beyond qualitative material selection toward quantitatively optimized operation and process protocols. First, pulse duration and frequency control may provide an effective route for minimizing unnecessary field exposure while allowing controlled ferroelectric activation during the initial wake‐up stage. Because oxygen‐vacancy redistribution, charge injection, and trap filling depend not only on the number of cycles but also on the effective electric‐field application time, pulse width, duty ratio, and cycling frequency should be optimized to achieve complete switching without excessive defect drift or leakage‐current accumulation.

Second, voltage‐step or adaptive conditioning protocols offer a promising approach for avoiding abrupt charge injection and local field concentration. Instead of applying a high switching voltage from the beginning, gradual voltage‐step cycling can induce controlled wake‐up, reduce interfacial charge accumulation, and suppress premature leakage‐path formation. The operating field should be selected within a stack‐specific optimized window, benchmarked relative to the *E_c_*, where complete polarization switching is achieved while charge injection, fatigue, and dielectric breakdown are minimized.

Third, systematic tuning of ALD‐level process parameters represents an important opportunity for controlling defect chemistry and phase stability. Precursor dosage, purge duration, oxidant dose, dopant‐insertion cycle, super‐cycle ratio, and precursor sequence can influence film stoichiometry, oxygen‐vacancy concentration, interfacial redox state, and atomic‐scale Hf/Zr mixing. Combining such ALD‐process control with electrode/interlayer engineering and multilayer structural design provides a practical roadmap toward wake‐up‐free, fatigue‐resistant, and high‐endurance HfO_2_‐based ferroelectric devices.

Beyond HfO_2_‐based ferroelectrics, the insights developed in this field are expected to provide a broadly applicable foundation for understanding and designing emerging ferroelectric material systems. Key physical factors governing wake‐up, fatigue, and endurance in HfO_2_, such as defect dynamics, phase stability, and interface‐controlled processes, are increasingly recognized as general determinants of ferroelectric reliability. Recent reports of ferroelectricity in systems such as atomic‐scale ferroelectric ZrO_2_ [187], where dimensionality‐driven phase transitions govern polarization stability, non‐oxide AlScN [188], where interface engineering enables thickness scalability under CMOS‐compatible conditions, and ultrathin TiO_2_ films [189], where dimensionality stabilizes a polar orthorhombic phase, suggest that these principles extend beyond HfO_2_‐based systems. Therefore, the fundamental understanding presented in this review provides a useful guideline for the development and optimization of next‐generation ferroelectric materials and devices. Future reliability studies should therefore combine defect/phase characterization with time‐resolved switching measurements using the finite‐pulse descriptors summarized in Table 4. Such measurements will enable quantitative correlation between defect redistribution, charge trapping, phase evolution, and practical switching dynamics.

Ultimately, continued progress in ferroelectric technologies will require an integrated approach that links defect chemistry, phase stability, interface engineering, processing conditions, and device operation. As device dimensions continue to shrink and application requirements become increasingly demanding, quantitative control of wake‐up and fatigue will be essential for achieving reliable long‐term operation. The mechanistic understanding and reliability‐design principles summarized in this review are expected to support the development of robust, scalable, and application‐ready ferroelectric materials and devices for future memory, logic, sensing, and neuromorphic systems.

## Author Contributions

**Hongseok Kim**: investigation, writing – original draft, writing – review and editing, visualization, data curation. **Shinhyeong Lee**: visualization, investigation, writing – original draft, writing – review and editing, data curation. **Minseung Park**: investigation, visualization. **Hyunah Cho**: investigation. **Hyojun Choi**: investigation, writing – original draft, writing – review and editing, visualization, data curation. **Yunseok Kim**: conceptualization, investigation, funding acquisition, writing – original draft, writing – review and editing, supervision, resources, data curation, project administration. **Min Hyuk Park**: conceptualization, investigation, writing – original draft, writing – review and editing, project administration, resources, supervision, data curation.

## Conflicts of Interest

The authors declare no conflicts of interest.

## Data Availability

Data sharing not applicable to this article as no datasets were generated or analysed during the current study.
